# Species delimitation in northern European water scavenger beetles of the genus *Hydrobius* (Coleoptera, Hydrophilidae)

**DOI:** 10.3897/zookeys.564.6558

**Published:** 2016-02-16

**Authors:** Erlend I. Fossen, Torbjørn Ekrem, Anders N. Nilsson, Johannes Bergsten

**Affiliations:** 1 Department of Biology, Centre for Biodiversity Dynamics, NTNU Norwegian University of Science and Technology, 7491 Trondheim, Norway; 2 Department of Natural History, NTNU University Museum, 7491 Trondheim, Norway; 3 Department of Ecology and Environmental Science, University of Umeå, S-901 87 Umeå, Sweden; 4 Department of Zoology, Swedish Museum of Natural History, Box 50007, SE-10405 Stockholm, Sweden

**Keywords:** GMYC, species complex, BPP, guide tree, Fennoscandia, morphometrics, Bayesian, genitalia, molecular phylogeny, species boundaries, morphology, cryptic species, integrative taxonomy, DNA barcoding, identification key, taxonomy, checklist

## Abstract

The chiefly Holarctic *Hydrobius* species complex (Coleoptera, Hydrophilidae) currently consists of *Hydrobius arcticus* Kuwert, 1890, and three morphological variants of *Hydrobius fuscipes* (Linnaeus, 1758): var. *fuscipes*, var. *rottenbergii* and var. *subrotundus* in northern Europe. Here molecular and morphological data are used to test the species boundaries in this species complex. Three gene segments (COI, H3 and ITS2) were sequenced and analyzed with Bayesian methods to infer phylogenetic relationships. The Generalized Mixed Yule Coalescent (GMYC) model and two versions of the Bayesian species delimitation method BPP, with or without an *a priori* defined guide tree (v2.2 & v3.0), were used to evaluate species limits. External and male genital characters of primarily Fennoscandian specimens were measured and statistically analyzed to test for significant differences in quantitative morphological characters. The four morphotypes formed separate genetic clusters on gene trees and were delimited as separate species by GMYC and by both versions of BPP, despite specimens of Hydrobius fuscipes var. fuscipes and Hydrobius fuscipes var. subrotundus being sympatric. *Hydrobius arcticus* and Hydrobius fuscipes var. rottenbergii could only be separated genetically with ITS2, and were delimited statistically with GMYC on ITS2 and with BPP on the combined data. In addition, six or seven potentially cryptic species of the *Hydrobius fuscipes* complex from regions outside northern Europe were delimited genetically. Although some overlap was found, the mean values of six male genital characters were significantly different between the morphotypes (p < 0.001). Morphological characters previously presumed to be diagnostic were less reliable to separate Hydrobius fuscipes var. fuscipes from Hydrobius fuscipes var. subrotundus, but characters in the literature for *Hydrobius arcticus* and Hydrobius fuscipes var. rottenbergii were diagnostic. Overall, morphological and molecular evidence strongly suggest that *Hydrobius arcticus* and the three morphological variants of *Hydrobius fuscipes* are separate species and *Hydrobius rottenbergii* Gerhardt, 1872, **stat. n.** and *Hydrobius subrotundus* Stephens, 1829, **stat. n.** are elevated to valid species. An identification key to northern European species of *Hydrobius* is provided.

## Introduction

The chiefly Holarctic genus *Hydrobius* Leach, 1815 (Hydrophilidae, Hydrophilinae) has nine species (Short and Fikáček 2011), including *Hydrobius orientalis* Jia and Short, 2009, recently described from a part of China belonging to the Oriental Region. The recent study of hydrophilid phylogeny made by Short and Fikáček (2013) indicated that *Hydrobius* as currently delimited in fact may be paraphyletic. The morphologically variable and strictly Holarctic *Hydrobius fuscipes* (Linnaeus, 1758) is seemingly closely related to the two genera *Ametor* Semenow, 1900, and *Sperchopsis* LeConte, 1861, known from North America, the East Palearctic and adjacent parts of the Oriental Region. The Nearctic *Hydrobius melaenus* (Germar, 1824), representing the more convex and less elongate species, was not close to *Hydrobius fuscipes* but had a more uncertain and not well supported placement within Hydrobiusini.

The circumpolar *Hydrobius fuscipes* group poses some severe problems when it comes to species delimitation, by tradition paid most attention to in West Europe so far, but including also three named species in the East Palearctic. In Europe only the two species, *Hydrobius fuscipes* and *Hydrobius arcticus* Kuwert, 1890, are recognized in current taxonomic works (de Jong 2011; Hansen 1987; Löbl and Smetana 2004).

Traditionally, however, three morphological variants of *Hydrobius fuscipes* have been recognized in Europe: Hydrobius fuscipes var. fuscipes, Hydrobius fuscipes var. subrotundus Stephens, 1829 and Hydrobius fuscipes var. rottenbergii Gerhardt, 1872. These taxa have different distributions. Hydrobius fuscipes var. rottenbergii is distributed in coastal areas of southern and central parts of Fennoscandia and Central Europe, Hydrobius fuscipes var. subrotundus is known from Fennoscandia and Central Europe, while Hydrobius fuscipes var. fuscipes has the largest distribution and is found in large parts of the Holarctic region. *Hydrobius arcticus* is distributed in the northern parts of Fennoscandia and European Russia (Hansen 1987; 1991). The taxa also have different habitat preferences with *Hydrobius arcticus* being a typical tundra species and Hydrobius fuscipes var. rottenbergii inhabiting rock pools with brackish water or rain water near tidal zones. Hydrobius fuscipes var. subrotundus and Hydrobius fuscipes var. fuscipes have more similar, but yet distinct, habitat preferences where the former prefer colder and more shady habitats and is often found in more acidic waters and near edges of running water. Hydrobius fuscipes var. fuscipes seems to prefer sun-exposed eutrophic stagnant ponds and can be found in temporary ponds and pools in open landscape (Hansen 1987). Despite different habitat preferences, Hydrobius fuscipes var. fuscipes can be found living in sympatry with *Hydrobius arcticus* in northern parts of Fennoscandia, and in sympatry with Hydrobius fuscipes var. subrotundus in parts of their common distribution range. Hydrobius fuscipes var. rottenbergii has on the other hand not been found in sympatry with the other species and variants (Balfour-Browne 1910; Hansen 1987; Schneider 1907).

The different variants of *Hydrobius fuscipes* have previously been considered separate species, but based on morphological studies that view has changed over time (e.g. Balfour-Browne 1910; 1958; Kuwert 1890; Rey 1885; Seidlitz 1891; Stephens 1839). All morphological variants were originally described as new taxa on the species-level, but a variable degree of synonymization has later occurred. *Hydrobius fuscipes* has more than 20 synonyms worldwide (Hansen 1999; Löbl and Smetana 2004), where only Hydrobius fuscipes var. rottenbergii, Hydrobius fuscipes var. subrotundus and Hydrobius fuscipes var. fuscipes currently are considered different enough to be regarded as distinct morphological variants (Hansen 1987). *Hydrobius arcticus* has fewer species synonyms worldwide, but was earlier considered as a morphological variant or as a subspecies of *Hydrobius fuscipes* (Hansen 1999).

The most recent study of the species complex involved morphological studies of approximately 400 specimens from Sweden and Finland and argued that the three variants of *Hydrobius fuscipes* are separate species based on morphological differences (Lindberg 1943). However, Lindberg (1943) did not include *Hydrobius arcticus* in his study and this makes his results and subsequent conclusion inadequate (Hansen 1987). Because of this, Hansen (1987) treated Hydrobius fuscipes var. subrotundus and Hydrobius fuscipes var. rottenbergii as intraspecific variation of *Hydrobius fuscipes*. This was later implemented in the world catalogue of Hydrophilidae (Hansen 1999) and in the catalogue of Palearctic Coleoptera (Löbl and Smetana 2004). No secondary sexual characters have been described in *Hydrobius*, and comparative genitalia studies have never been conducted on the northern European species (Balfour-Browne 1910; Hansen 1987).

Species-level documentation of biological diversity and analyses of species boundaries have increased with the availability of genetic data and new methodological approaches (Carstens et al. 2013). While many morphological studies delimit species by use of discrete characters or continuous quantitative characters without overlap between species, both quantitative body- and male genitalia characters have been used to delimit species within species complexes of beetles (e.g. Bergsten et al. 2012b; Drotz et al. 2001; Nilsson 1987; 1994; Nilsson and Ribera 2007; Tocco et al. 2011). Usually the molecular loci used in species delimitation studies are neutral markers and not directly involved in the actual emergence of reproductive barriers between incipient species. Molecular methods developed for identification purposes like a 10x barcode-gap threshold (Hebert et al. 2004) are clearly inadequate for some organism groups, especially as it fails to recognize young species (Hickerson et al. 2006). Also the expectation of reciprocal monophyly in genealogies has limitations as the process of lineage sorting can take considerable time and is dependent on the effective population size (Bergsten et al. 2012a). Recently, more sophisticated statistical methods have been developed to delimit species using molecular data. These methods can be categorized into two groups based on whether or not sample assignment is required (Carstens et al. 2013). Discovery methods are methods where data are analyzed without *a priori* partitioning of samples. Validation methods, however, require *a priori* partitioning of samples and should only be used in situations where either existing knowledge of the taxonomy or other characters can be used to make a testable hypothesis for delimitation, or where populations are clearly delineated (Carstens et al. 2013).

The Generalized Mixed Yule Coalescent (GMYC) model (Pons et al. 2006) is a discovery method that applies the phylogenetic species concept with assumed reciprocal monophyly in gene trees. It has increasingly been used in recent times to delimit closely related species (e.g. Cornils and Held 2014; Hjalmarsson et al. 2013; Pardo et al. 2014; Rodriguero et al. 2013; Zhang et al. 2014). Analyses are based on ultrametric single-locus genealogies as input, where the rate of branching is expected to be higher between specimens of the same species than between specimens of different species. The method attempts to model the transition point where there is a shift in the branching rate. This shift reflects the transition from between-species processes (e.g. speciation and extinction) to within-species processes (coalescence).

The Bayesian species delimitation method BPP (Bayesian Phylogenetics and Phylogeography) as originally presented is a validation method that applies reversible jump Markov chain Monte Carlo iterations (rjMCMC) to estimate the posterior probability of different hypotheses of species delimitation (Rannala and Yang 2003; 2013; Yang and Rannala 2010; 2014). The method estimates ancestral population sizes (within species) and species divergence times (between species) and can be used in species delimitation using multi-locus sequence data from closely related species. It required a guide tree as input in earlier versions (e.g. BPP v2.2), in which a species tree where the topology and the assignment of terminals into proposed species, are defined before analysis. However, version 3.0 (Yang and Rannala 2014) has overcome the need for a guide tree and estimates the species tree with a Nearest-Neighbor Interchange (NNI) algorithm simultaneously as species are delimited. This is a significant advantage over the old version since misspecifications of the guide tree can affect how many species are delimited and give misleading results (Leache and Fujita 2010). In principal if each specimen is assigned to a separate population, BPP version 3.0 also makes redundant the *a priori* assignment of specimen to (maximally subdivided) potential species and truly becomes a discovery method (Yang and Rannala 2010; 2014). However, such analyses are discouraged, except for very small datasets, because of the size of parameter space and computational complexity (Yang and Rannala 2014). The species delimitation algorithm computes the posterior probabilities of each node in the evaluated species tree (or guide tree in older versions) representing a speciation event by allowing the rjMCMC to sample all the possible ways of collapsing nodes in the species tree (or guide tree) into fewer species. BPP uncouples gene trees and species trees and therefore has the benefit of allowing the gene tree coalescences to be older than species tree coalescences. This accommodates the issue of gene trees and species trees often not being the same (Rannala and Yang 2003; 2013; Yang and Rannala 2010; 2014). BPP is increasingly used to delimit species (e.g. Bochkov et al. 2014; De Crop et al. 2014; Derkarabetian and Hedin 2014; Guillin et al. 2014; Hamback et al. 2013), but as of to date few studies have used the guide tree-free BPP v3.0 on empirical data.

The mitochondrial gene cytochrome c oxidase subunit I (COI) is the standard genetic marker used to identify animal species with DNA Barcoding (Hebert et al. 2003). High substitution rates and deep divergences between closely related species in many animal groups have contributed in making COI the primary marker for the Barcode of Life Initiative. However, mitochondrial DNA (mtDNA) is maternally inherited in insects, thus occurrence of heteroplasmy (e.g. Magnacca and Brown 2010), male-killing or cytoplasmic incompatibility-inducing symbionts (e.g. *Wolbachia*; Werren et al. 2008) or introgressive hybridization (Ballard and Whitlock 2004) can produce misleading results in conflict with patterns based on nuclear DNA (e.g. Shaw 2002). Because of this, it is an advantage to use both mitochondrial and nuclear loci when analyzing species boundaries.

The main objective of this study was to statistically test species boundaries in the northern European *Hydrobius fuscipes* group using both molecular (three gene segments: COI, H3 and ITS2) and morphological data (both external and male genital characters).

## Material and methods

### Specimens

For the sake of simplicity, *Hydrobius arcticus* and the different variants of *Hydrobius fuscipes* will from here on be referred to as “morphotypes” and listed with subspecies terminology.

Adult specimens of the four morphotypes were obtained from expeditions throughout the Palearctic and Nearctic regions, with the most extensive sampling being in Norway and Sweden. The specimens were collected at various localities using an aquatic net in shallow vegetation along the edges of lakes, ponds and pools. The specimens were immediately stored in 70–96% ethanol after capture to keep optimal preservation conditions. Additional specimens from the Palearctic and Nearctic regions were obtained on loan from natural history museums and other institutions in Europe (Table S1 in Suppl. material 3). Type specimens of the different species and variants were borrowed and examined morphologically when possible, but we were unable to examine the type of *Hydrobius arcticus* (Table 1). The type of *Hydrobius fuscipes* was not examined, but the Linnean Society of London made an image available for examination. Specimens used in DNA extraction were dried and glued on mounting cards after measurements were taken. Specimens were identified with the use of appropriate identification keys and diagnostic characters (Hansen 1987).

**Table 1.** Examined type specimens of *Hydrobius*. ^†^ Specimen not examined, an image of the specimen was used in morphological analyses.

Variant of *Hydrobius fuscipes*	Type	Type locality	Storing institution
*fuscipes* (Linnaeus, 1758)	Holotype^†^	Europe	Linnean Society of London, UK
*subrotundus* Stephens, 1829	Possible syntype	British Isles	Natural History Museum, London, UK
*rottenbergii* Gerhardt, 1872	3x syntypes	Germany or Poland	Bavarian State Collection of Zoology, Munich, Germany

In total, 62 *Hydrobius arcticus*, 100 *Hydrobius fuscipes subrotundus*, 97 *Hydrobius fuscipes rottenbergii* and 130 *Hydrobius fuscipes fuscipes* specimens were examined in this study. The specimens used were chosen pseudo-randomly depending on distribution and availability with the intent to cover all morphotypes from most of their distribution area with a clear focus on the morphotypes of *Hydrobius* in northern Europe. Detailed morphological measurements and molecular analyses were conducted on a subsample of these specimens (approximately 30 of each morphotype, Suppl. material 1).

### DNA extraction, amplification and sequencing

Most specimens used in the molecular analyses were relatively fresh (0-11 years old) and stored in 70-96% ethanol prior to the extraction; the oldest successfully extracted specimens had been pinned for 15 years before extraction. Whole specimens were used to extract DNA, but lysis was done non-destructively to preserve the exoskeleton for morphological analysis. The second or third abdominal ventrite of the specimens was punctured with sharp sterile forceps to facilitate lysis and diffusion of DNA out of the specimens. The forceps were cleaned between handling of different specimens with DNA AWAY™ Surface Decontaminant (Thermo Scientific, Wilmington, USA) and 80% ethanol. Beetles were placed in 100 µL Lysis Buffer (Mole Genetics, Lysaker, Norway) and 4 µL QIAGEN® Proteinase-K (QIAGEN, Venlo, Netherlands) and incubated overnight at 56 °C for 7-12 hours. The lysate was transferred to sample tubes after lysis and MoleStrips^TM^ DNA Tissue (Mole Genetics) was used to extract DNA using a GeneMole® robot (Mole Genetics). Either 100 µL or 200 µL elution buffer was used for elution; 100 µL elution buffer used for older specimens. A selection of the specimens (n = 5) went through the DNA extraction process twice to be used as controls.

Three presumed unlinked gene segments were analyzed, one protein-coding mitochondrial gene segment (COI), one protein-coding nuclear gene segment (Histone H3; abbr. H3), and one non-functional nuclear rDNA segment (Internal transcribed spacer 2; abbr. ITS2) (Table 2). Each PCR reaction mixture contained 2 or 3µl DNA template (3µl for concentrations < 10 ng/µl, else 2µl), 1 µl of forward and reverse primer (10µM), a mixture with Taq polymerase, and molecular grade water (ddH_2_O) for a total reaction volume of 25µl. Two different Taq polymerase mixtures were used: HotStarTaq® DNA Polymerase (QIAGEN) and premixed illustra^TM^ puReTaq Ready-To-Go PCR Beads (GE Healthcare, Uppsala, Sweden). The HotStarTaq® mixture contained 2.5µl 10x PCR-buffer, 2.0µl MgCl_2_ (25mM), 2.0µl dNTPs (5mM each) and 0.2 µl HotStarTaq® DNA Polymerase. While both reaction mixtures were able to successfully amplify the gene segments, the Ready-To-Go PCR Beads had a higher success rate than the HotStarTaq® mixture for all gene segments.

**Table 2.** Primers used in PCR and sequence reactions.

**Gene**	**Forward primer**	**Sequence**	**Reference**
COI	LCO1490	5’-GGTCAACAAATCATAAAGATATTGG-3’	Folmer et al. (1994)
H3	HexAF	5’-ATGGCTCGTACCAAGCAGACGGC-3’	Ogden and Whiting (2003)
ITS2	CAS5p8sFc	5’-TGAACATCGACATTTYGAACGCACAT-3’	Ji et al. (2003)
			
**Gene**	**Reverse primer**	**Sequence**	**Reference**
COI	HCO2198	5’-TAAACTTCAGGGTGACCAAAAAATCA-3’	Folmer et al. (1994)
H3	HexAR	5’-ATATCCTTGGGCATGATGGTGAC-3’	Ogden and Whiting (2003)
ITS2	CAS28sB1d	5’-TTCTTTTCCTCCSCTTAYTRATATGCTTAA-3’	Ji et al. (2003)

All PCR reactions were performed with a C1000^TM^ Thermal Cycler (Bio-Rad Laboratories, Foster City, USA). Blank samples with molecular grade water (ddH_2_O) instead of DNA template were used as control-samples in all PCR-runs. The following PCR conditions were used in the amplification of the COI Barcode segment with the HotStarTaq® mixture: initial denaturation for 5 min at 95 °C; 60 s at 94 °C; 5 cycles of 30 s at 94 °C, 30 s at 45 °C, 60 s at 72 °C; 35 cycles of 30 s at 94 °C, 30 s at 51 °C, 60 s at 72 °C; ending with a final elongation for 5 min at 72 °C. Amplification of the COI Barcode segment with the Ready-To-Go PCR Beads: initial denaturation for 5 min at 95 °C; 42 cycles of 30 s at 95 °C, 30 s at 45 °C, 60 s at 72 °C; ending with a final elongation for 8 min at 72 °C. Amplification of H3 with HotStarTaq® mixture and Ready-To-Go PCR Beads: initial denaturation for 5 min at 95 °C; 40 cycles of 30 s at 95 °C, 30 s at 50 °C, 60 s at 72 °C; ending with a final elongation for 8 min at 72 °C. Amplification of ITS2 with HotStarTaq® mixture and Ready-To-Go PCR Beads: initial denaturation for 5 min at 95 °C; 35 cycles of 40 s at 94 °C, 30 s at 55 °C, 40 s at 72 °C; ending with a final elongation for 7 min and 45 s at 72 °C.

Aliquots of the PCR-products selected for sequencing were purified with illustra^TM^ ExoStar^TM^ 1-Step (GE Healthcare) or with illustra^TM^ ExoProStar^TM^ 1-Step (GE Healthcare) following the producers recommendation. Samples were sequenced in both directions by cycle sequencing technology using dideoxy chain termination/cycle sequencing on ABI 3730XL sequencing machines at Eurofins Genomics (Germany).

In cases where DNA was extracted twice from the same specimens, both replicates were sequenced if successfully amplified with PCR. The replicates were used as controls and were expected to yield the same sequence.

Sequenced specimens are kept as DNA vouchers at their respective institutions, labeled with the IDs listed in Suppl. material 1.

### Molecular analysis

**Editing and alignment of sequences**

DNA Baser Sequence Assembler v4.10.1.13 (2012, Heracle BioSoft SRL, http://www.DnaBaser.com) was used to assemble and edit DNA sequences. The forward and reverse sequences were automatically assembled by the software and the contig was inspected and edited manually. When base calls were ambiguous, the appropriate International Union of Pure and Applied Chemistry (IUPAC) codes were used to represent this. In a few cases the chromatogram was only readable in one direction. Sequences with very low quality were not used in downstream analysis.

Sequences are available in the BOLD project FENHY (http://www.boldsystems.org/index.php/MAS_Management_OpenProject?code=FENHY) and submitted to GenBank under accession numbers KU380492–KU380737. Additional COI Barcodes were also downloaded from BOLD (Ratnasingham and Hebert 2007) and used in downstream analyses (Suppl. material 1), including sequences from Hendrich et al. (2015) and Pentinsaari et al. (2014). The following acronyms were used for the geographical locations of the samples in the phylogenetic trees: CAN = Canada, FIN = Finland, GER = Germany, GREECE = Greece, ITA = Italy, NOR = Norway, POR = Portugal, RUS = Russia, SPA = Spain, SWE = Sweden, UK = United Kingdom, and USA = United States of America.

MEGA v6.06 (Tamura et al. 2013) was used to align the edited nucleotide contigs. All segments were aligned with MUSCLE (Edgar 2004) under default settings, where the COI and H3 segments were aligned as amino acids, whereas ITS2 was aligned as DNA. The ends of all three alignments were trimmed to remove low quality parts of sequences and primers. BLAST (Altschul et al. 1990) was used on irregular sequences to identify and remove contaminants.

### Phylogenetic analyses

Bayesian methods were used to find the phylogenetic relationship between specimens of different morphotypes. Analyses of both single locus datasets and a concatenated dataset were conducted. The concatenated dataset combined all three gene segments (COI, H3 and ITS2), removing any samples that lacked sequences from one or two genes to avoid large sections of missing data in the matrix. *Hydrobius convexus* was used as outgroup in all phylogenetic analyses.


 Bayesian information criterion (BIC) was used within PartitionFinder v1.1.1 (Lanfear et al. 2012) to find and select the best fit substitution model and partition scheme for use in Bayesian analyses.

MrBayes v3.2 (Ronquist et al. 2012) was used for Bayesian phylogenetic inference of sequence data. The best partition schemes and corresponding substitution models from PartitionFinder were used in two simultaneous but independent analyses using Metropolis-coupled Markov chain Monte Carlo (MCMCMC) iterations each with four chains (nchains = 4). The number of generations run for each analysis was dependent on the size of the dataset and whether or not convergence was easy to obtain, but a minimum of 2,500,000 generations were always run (ngen ≥ 2,500,000). Heating of chains was set to 0.2 (temp = 0.2). Sampling frequency was set to every 1000 generation (samplefreq = 1000). Trace plots were used to determine the required burnin and the first 25% of sampled trees were discarded as burn-in trees (relburnin = yes burninfrac = 0.25). Standard deviation of split frequencies (≤ 0.01), effective sample sizes (ESS) and trace plots visualized with Tracer v1.6 (Rambaut et al. 2013) were used as convergence diagnostics. A 50% majority rule consensus tree (contype = halfcompat) was calculated from the remaining sampled trees after the removal of burn-in.

### Species delimitation

The maximum likelihood based GMYC model (Pons et al. 2006) and the Bayesian method applied in BPP v3.0 and BPP v2.2 (Rannala and Yang 2013; Yang and Rannala 2010; 2014) were used to evaluate species delimitations.

The GMYC analyses were conducted in the statistical software R v3.0.3 (R Core Team 2014), with the use of ape, MASS, gee, paran and splits packages. The input for the GMYC was an ultrametric single locus gene tree with multiple individuals per species for multiple potential species. To test if a strict molecular clock could be appropriate to infer the ultrametric trees, stepping-stone sampling was used in MrBayes v3.2 (Ronquist et al. 2012) to find the marginal model likelihoods for a model with a strict molecular clock and for a time-free model. The tests were run 5 times for each model and averages of these runs were used to compare the models in a Bayes factor test. The marginal likelihood of the models with a strict molecular clock were higher for all three gene segments than the time-free models, thus implementing a strict molecular clock was justified.

The ultrametric trees, one for each gene segment, were made with BEAST v2.1.3 and corresponding user interface (BEAUti 2) (Bouckaert et al. 2014). The best partition schemes and corresponding substitution models found in PartitionFinder were used with sites unlinked, while the clock and tree models were linked. A strict clock model was implemented and a Coalescent Constant Population prior was used as the tree prior. The numbers of generations were 10 million for H3 and ITS2 data and 20 million for COI data. Sampling of parameters and trees was set to every 1000 (H3 and ITS2 data) or 2000 (COI) generations. Effective sample sizes (ESS) and trace plots estimated with Tracer v1.6 (Rambaut et al. 2013) were used as convergence diagnostics. Sampled trees from two independent runs were pooled together after manually discarding 15% (H3 and ITS2) or 20% (COI) of the trees as burn-in (determined by examining trace plots). Ultrametric maximum clade credibility (MCC) trees were computed using the mean node heights with TreeAnnotator v2.0.3 (Drummond and Rambaut 2007) for each gene segment. The arbitrary time scales of the trees were rescaled so that the root had an age of 1.

The GMYC analyses were conducted with the single-threshold version, since Fujisawa and Barraclough (2013) found it to outperform the multiple-thresholds version on simulated data. The maximum likelihood of the GMYC model was tested with a likelihood ratio test against a one-species null model (where the entire tree is considered as a single coalescent).

Comparison and selection of the best models were performed with the method described by Powell (2012), where Akaike Information Criterion values taking sample size into account (AICc) of the different models are compared. Models with Δ AICc-values from 0 to 2 are considered the best explanations of the data among the models compared, models with Δ AICc-values from 4 to 7 are generally considered to have little support from the data, whereas models with Δ AICc-values >10 are considered to have essentially no support from the data compared to the other models (Burnham and Anderson 2002). Support values of the GMYC-delimited species (GMYC-support; Fujisawa and Barraclough 2013), defined as the sum of Akaike weights of candidate delimitation models in which a specific node is included, were calculated using models within the 95% confidence set.

The Bayesian species delimitation methods in BPP v3.0 and BPP v2.2 (Rannala and Yang 2013; Yang and Rannala 2010; 2014) were used with multi-locus data (COI, H3 and ITS2). All analyses included *Hydrobius convexus* as outgroup, as Rannala and Yang (2013) showed that including a closely related outgroup may increase the statistical power of BPP. Five different species scenarios with a total of 4 guide trees were used in BPP v2.2. The assignment of specimens to potential species for both BPP versions, and the topologies used in the guide trees in BPP v2.2, were chosen based on taxonomical knowledge (from morphological studies), the species delimited with GMYC and based on the topology and clusters found in the phylogenetic trees. The four known northern European morphotypes of *Hydrobius* were the main focus of the species delimitation tests.

Each theta (*Θ*, ancestral population size) and tau (*τ*, species divergence time) parameters in the BPP analyses (both versions) used priors specified with a gamma distribution with mean α/β. Only the root in the species tree (*τ*_0_) was given as a tau prior whereas other *τ* parameters were generated with the Dirichlet distribution with default settings in BPP. α = 1 was used as a diffuse prior in all analyses, while different combinations of β were tested for *Θ* and *τ*_0_. Multiple initial runs with different combinations of β were used to find combinations of β that made the means (α/β) be within an order of magnitude from the posterior estimates of *Θ* and *τ*_0_, as recommended by Zhang et al. (2011). The dataset had a posterior estimate of *Θ* ≈ 0.01 and a posterior estimate of *τ*_0_ ≈ 0.03. The following four combinations of gamma distributions were used in both BPP versions; 1: *Θ*: G(1, 50), *τ*_0_: G(1, 20); 2: *Θ*: G(1, 50), *τ*_0_: G(1, 200); 3: *Θ*: G(1, 500), *τ*_0_: G(1, 20); and 4: *Θ*: G(1, 500), *τ*_0_: G(1, 200). The combinations include the posterior estimates of *Θ* and *τ*_0_ and the means (α/β) are within an order of magnitude of these estimates.

All BPP-analyses were run for 100,000 generations with sampling every two generations (nsample = 50,000 and sampfreq = 2), after discarding an initial burn-in of 40,000 generations (burnin = 40,000). Heredity scalars were set to 0.25 for COI and 1.0 for H3 and ITS2. Automatic adjustments of finetune parameters were used while making sure that the acceptance proportions were within the range of 0.2–0.7 as recommended by Yang and Rannala (2010). Every analysis was run twice with different starting species trees to check for convergence between runs and agreement on the posterior probability of the species delimitation models. Both algorithm “0” and algorithm “1” (see Yang and Rannala 2010) were tested and gave very similar results, and thus primarily results obtained with algorithm 0 will be reported.

### Morphological analysis

Specimens were examined with a Leica MZ16 stereomicroscope (Leica Microsystems, Wetzlar, Germany) in reflected light using the measurement module of the software Leica Application Suite 3.2 (Leica Microsystems).

Detailed morphological measurements were conducted after results from the molecular analyses were obtained. A total of 21 *Hydrobius arcticus*, 33 *Hydrobius fuscipes subrotundus*, 26 *Hydrobius fuscipes rottenbergii* and 33 *Hydrobius fuscipes fuscipes* specimens were measured, selected primarily based on the presence of molecular data, to link morphological and molecular divergence patterns. Some specimens that were not included in the molecular analyses were also measured to increase the sample size, especially specimens of *Hydrobius arcticus* and *Hydrobius fuscipes rottenbergii*. These specimens were selected based on morphology and geographical locality, making sure they were of the correct species/variant. Characters that seemed to have very high intraspecific variation or were prone to high amounts of measurement errors were excluded from statistical analyses. The measurements of the first 10 specimens were repeated at a later stage to detect potential errors and ensure repeatability in measurements. A large selection of presumably diagnostic and informative external body characters were measured and analyzed. Genitalia were dissected in male specimens and genital characters examined and measured at approximately 60x magnification. A total of 15 *Hydrobius arcticus*, 16 *Hydrobius fuscipes subrotundus*, 15 *Hydrobius fuscipes rottenbergii* and 16 *Hydrobius fuscipes fuscipes* had their genitalia measured, including type specimens of *Hydrobius fuscipes rottenbergii* and *Hydrobius fuscipes subrotundus*. For pinned specimens, the genitalia were dissected after softening of the specimens in warm water for 10-20 minutes. A hooked needle was used to bring the genital capsule out from the abdomen, before the genitalia was separated from the genital capsule with two needles while placed in ethanol under a stereomicroscope. The abdomen and genitalia were placed on the same mounting card after measurements were conducted.

### Characters

**Figure 1.**
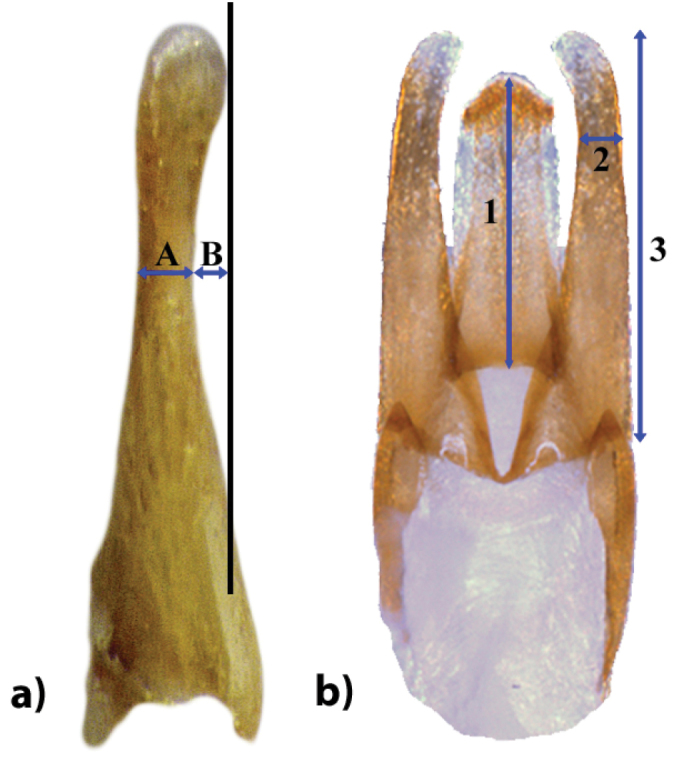
Measurements of *Hydrobius* male genitalia. **a** Paramere in lateral view. A: width of paramere (character 1.5). Curvature of paramere tip (character 1.6) = A+B **b** Genitalia in dorsal view. 1: Length of sclerotized part of penis. 2: Width of narrowest part of paramere (character 1.2). 3: Length of paramere (character 1.1). Robustness of paramere (character 1.3) = 3 / 2. Paramere length relative penis length (character 1.4) = 3/1. Images of *Hydrobius fuscipes rottenbergii*.

A total of 29 characters was examined and measured, 14 male genital characters and 15 external body characters (Suppl. material 2). The following six genital and four body characters were most informative:

**Male genital characters** (Fig. 1)

The mean of the left and right paramere character were used as one character for characters measured in dorsal view.

1.1) *Length of parameres*: dorsal view. Measured as the total length from the tip of the paramere to the bottom part of the paramere where it overlaps with the basal piece of the aedeagus.

1.2) *Width of parameres*: dorsal view. Measured as the width of the paramere at the narrowest part.

1.3) *Robustness of parameres*: dorsal view. Measured as a ratio between the lengths of the parameres (character 1.1) divided by the narrowest width of paramere (character 1.2). A low value means that the paramere is more robust.

1.4) *Ratio between paramere length and penis length*: dorsal view. Measured as the length of the paramere (character 1.1) divided by the length of the sclerotized part of the penis.

1.5) *Width of paramere*: lateral view. Measured as width of the paramere at the narrowest part.

1.6) *Curvature of paramere tip*: lateral view. Measured as length from dorsal side of the narrowest part of the paramere to a vertical line from the tip of paramere on the ventral side, parallel to the dorsal line.

**Figure 2.**
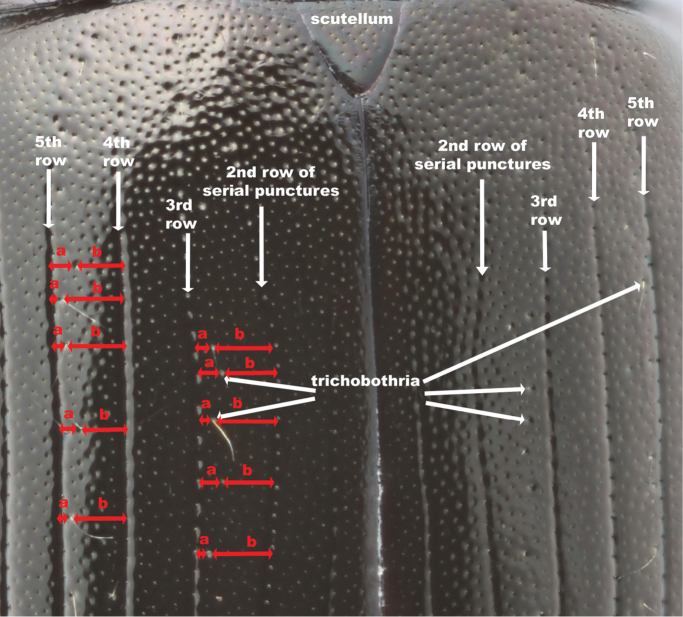
Measurement of the relative position of trichobothria on the elytra (character 2.1). Dorsal view of anterior part of the elytra, showing how several trichobothria encountered posterior to the scutellum were measured. Each relative position of a trichobothrium was measured by dividing the length from the 3^rd^ row of serial punctures to the trichobothrium (a) by the length from the 3^rd^ row to the 2^nd^ row (a+b). The same was done with trichobothria in or near the 5^th^ row of serial punctures. Image of *Hydrobius fuscipes fuscipes*.

**Body characters**:

2.1) *Relative position of trichobothria (systematic punctures) in relation to the 3rd and 5th row of elytral serial punctures*: previously used to separate variants of *Hydrobius fuscipes* (Hansen 1987). Quantified and measured as a ratio between the length from the 3th or 5th row of serial punctures to the first 20 trichobothria posterior to scutellum, divided by the length from the 3rd or 5th row to the 2nd or 4th row, respectively (Fig. 2). A low value means that the trichobothria are close to the 3rd or 5th row of serial punctures, while a higher value, e.g. 0.5, means that they are positioned in the elytral intervals.

**Figure 3.**
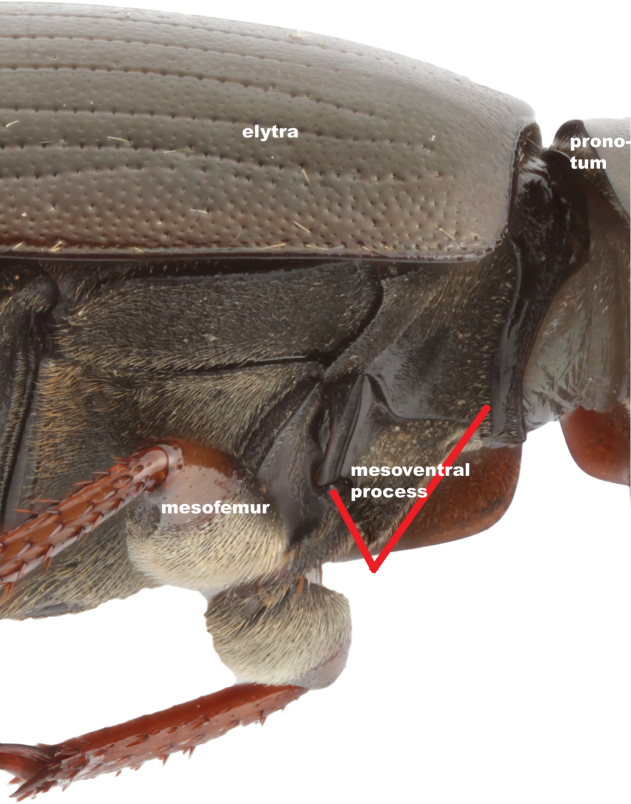
The shape of the mesoventral process (character 2.2). Measured in lateral view as an angle (indicated by red lines). Image of *Hydrobius fuscipes fuscipes*.

2.2) *Shape of mesoventral process*: previously used to separate *Hydrobius fuscipes* from *Hydrobius arcticus* (Hansen 1987). Measured in lateral view as an angle (Fig. 3). A low value means that the mesoventrite has a relatively strong acute process.

2.3) *Color of legs*: previously used to separate variants of *Hydrobius fuscipes* (Hansen 1987). The colors of the tibiae and femora were examined qualitatively.

**Figure 4.**
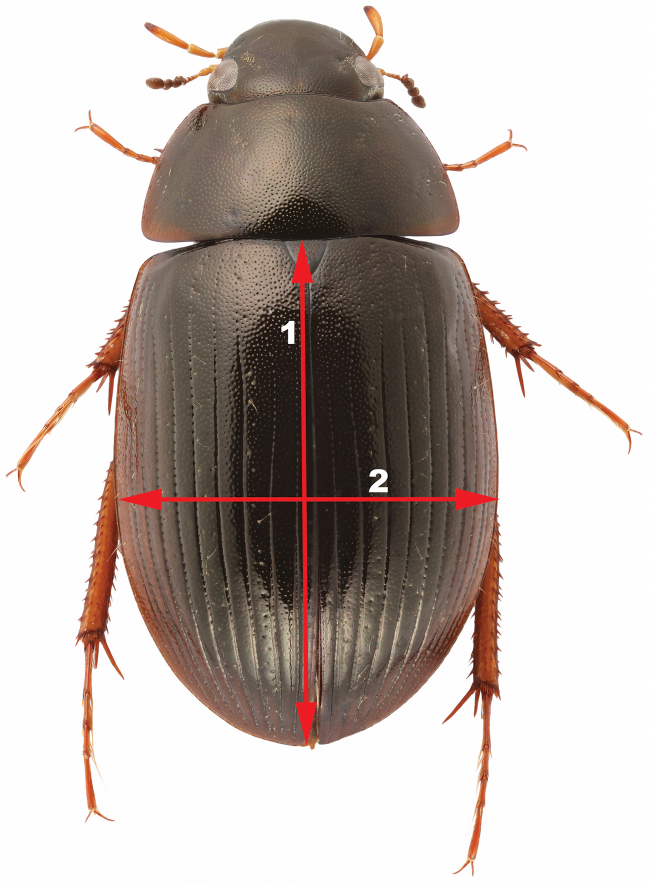
Measurement of Elytral Index (EI). **1** Length of elytra **2** Maximum width of elytra. EI (character 2.4) = 1 / 2. Image of *Hydrobius fuscipes fuscipes*.

2.4) *Body shape*: previously used to separate variants of *Hydrobius fuscipes* (Hansen 1987). Quantified with the *Elytral Index* (EI), where the length of the elytra is divided by the maximum width of the elytra, when both elytra are in focus (Fig. 4). A low value means that the body shape is shorter and more convex.

### Statistical analysis of morphological characters

In order to find a reliable estimate of body size, repeated measurements of the total body length, measured from the anterior margin of the labrum to the posterior elytral apex, were compared to the combined length of elytra and the length of pronotum in 19 specimens. The sum of the elytra and the pronotum lengths was found to be less variable between repeated measurements than the complete body length and was therefore used as a more reliable and reproducible estimate of body size in all analyses. A potential bias towards one side (left or right) of assumed symmetric characters was examined using a Student’s t-test to see if the means of right and left structures were statistically different. A visual comparison of the differences by using a histogram showing the differences between the left and right structure was also conducted.

To test if the morphotypes were significantly different in the measured characters, an analysis of covariance (ANCOVA) was used with log-transformed character values as the response variable, the morphotypes as a predictor variable and a log-transformed estimate of body size as a covariate. The estimated body size was used to control for any confounding allometric relationships between the morphological character and body size. The models were reduced, by comparing the models’ adjusted R^2 values and AIC-values, to only include statistically significant effects, including reduction to an analysis of variance (ANOVA) in cases where body size was non-significant. Post hoc comparison of the morphotypes was performed with Tukey’s HSD (honestly significant difference) test with adjusted p-values. Non-log-transformed variables were used in cases where the models without log-transformed variables had a greater R^2 value than the models with log-transformed variables. Characters that are ratios were not log-transformed, neither did body size in these analyses, as the allometric relationship for ratios are less predictable. A selection of interesting male genital characters were plotted against each other and a Convex Hull (de Berg et al. 2000) was used to illustrate the overlap of different morphotypes with regard to the characters of interest. All statistical analyses were performed with the statistical software R v3.0.3 (R Core Team 2014).

## Results

Additional tables (S2–S10) and figures (S1–S6) are available in Supplementary material 3.

### Molecular analyses

A total of 86 specimens from the four morphotypes was successfully sequenced for at least one gene segment (Table 3). Due to availability of fresh material, the number of successfully sequenced *Hydrobius arcticus* specimens (11) was considerably lower than the specimens of *Hydrobius fuscipes* variants (Table 3). There seem to be no clear differences in sequencing success among gene segments, but H3 amplified for a few more samples.

**Table 3.** Number of successfully sequenced gene segments from *Hydrobius* morphotypes. ^†^COI sequences from BOLD are not included.

Gene segment	Morphotype				Sum
	*Hydrobius arcticus*	*Hydrobius fuscipes fuscipes*	*Hydrobius fuscipes rottenbergii*	*Hydrobius fuscipes subrotundus*	
COI	7	29	14	30	80^†^
H3	9	30	14	31	84
ITS2	9	27	14	29	79
Specimens with at least one segment	11	30	14	31	86^†^
Specimens with all three segments	5	27	14	29	75

### Sequence composition and alignment

The alignments were unproblematic as there were very few insertions or deletions (indels) (Table 4). Neither COI nor H3 had any indels, whereas the ingroup had one indel of 2–4 bases for ITS2. COI was the most variable segment with 21.3% variable and 18.5% parsimony informative sites in the ingroup. H3 had 9.15% variable and 6.54% parsimony informative sites, while ITS2 had 6.94% variable and 6.68% parsimony informative sites (Table 4). The length of COI used in analyses was 1.5 to 2 times more than the other segments, and the number of unique haplotypes was also proportionally higher for COI compared to the other two segments (Table 4).

**Table 4.** Basic statistics on gene segments used in molecular analyses of the *Hydrobius* species complex. Unique sites refer to variable but parsimony uninformative sites. ^†^ Only specimens with all three gene segments were included in the concatenated dataset.

	**COI** (incl./excl. outgroup)	**H3** (incl./excl. outgroup)	**ITS2** (incl./excl. outgroup)	**Concatenated dataset** ^†^(incl./excl. outgroup)
Length of segment (bp)	658/658	328/328	405/405	1391/1391
Length used in analyses, incl. gaps (bp)	611/611	306/306	412/389	1329/1306
Indels in aligned segment	0/0	0/0	3/1	3/1
Conserved sites (bp)	446/481	247/278	338/362	1041/1131
Variable sites (bp)	165/130	59/28	51/27	265/175
Parsimony informative sites (bp)	116/113	22/20	26/26	154/149
Unique sites (bp)	49/17	37/8	25/1	111/26
A (%)	30.4/30.4	25.7/25.8	16.6/16.6	25.2/25.2
C (%)	17.3/17.3	30.9/30.9	29.6/29.6	24.1/24.1
G (%)	16.3/16.3	24.1/24.0	32.5/32.5	22.9/22.9
T (%)	36.0/36.0	19.3/19.3	21.3/21.4	27.8/27.8
Number of unique haplotypes	49/48	18/17	12/11	37/36

### Best fit substitution models and partition schemes

There was large agreement between the best partition schemes and substitution models for the single locus gene segments compared to the concatenated dataset, although for example codon position 3 of H3 is assigned a K80+I model when using H3 data and a K80 model when using the concatenated data (Table S2 in Suppl. material 3). Less complex substitution models were most fit when using the H3 and ITS2 datasets without outgroups than when outgroups were included (Table S2 in Suppl. material 3).

### Phylogenetic analyses

**Figure 5.**
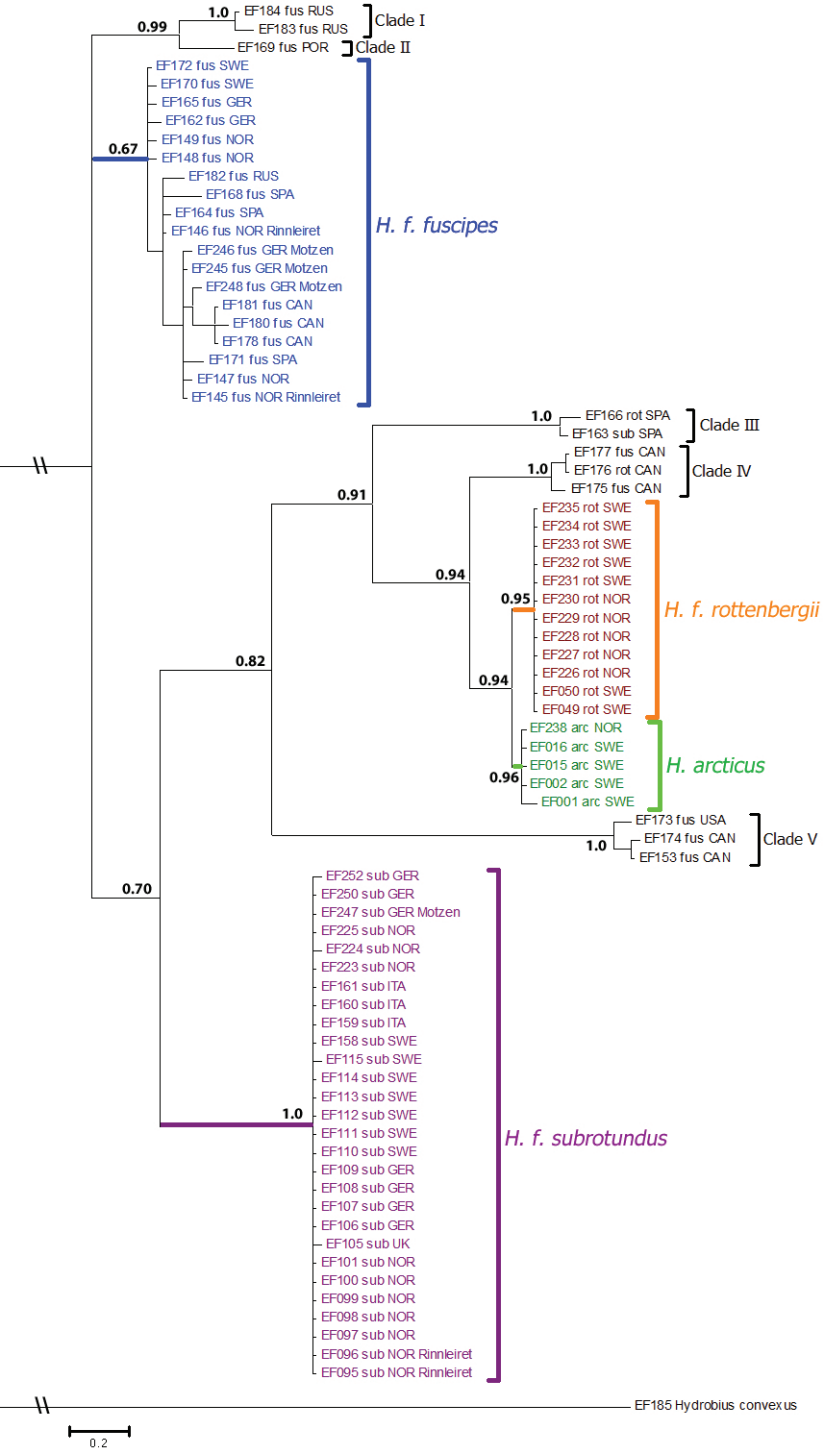
Majority-rule consensus tree from time-free Bayesian analysis of the concatenated data. Branch support values are posterior probabilities. Samples are labeled with ID-numbers, identified morphotypes and country of origin. Specimens collected in sympatry are also labeled with locality name (Rinnleiret or Motzen). Scale bar indicates expected number of nucleotide substitutions per site. Branches with “\\” have been manually cut. Abbreviations for morphotypes: arc = *arcticus*, fus = *fuscipes*, rot = *rottenbergii*, sub = *subrotundus*.

Up to eleven different genetically divergent clades, one of which is represented by a singleton, were found in the phylogenetic trees, although with different amount of consistency and support between the different gene segments analyzed. Highest resolutions were found in the trees resulting from analyses of COI and the concatenated dataset (Fig. 5 and Fig. S1 in Suppl. material 3), presumably as these datasets show the most variation. Some geographical structuring was found among the clades (Table 5). Within northern Europe, four clades (*Hydrobius arcticus*, *Hydrobius fuscipes rottenbergii*, *Hydrobius fuscipes fuscipes* and *Hydrobius fuscipes subrotundus*) are found, which correspond well with the respective described morphospecies. *Hydrobius fuscipes fuscipes* and *Hydrobius fuscipes subrotundus* have the widest distribution, whereas *Hydrobius arcticus* and *Hydrobius fuscipes rottenbergii* are only found in Norway and Sweden among included material. Clades I-III, VI and VII are central and southern European clades, whereas IV and V are North American clades (Table 5).

**Table 5.** Genetically divergent clades and their localities, including corresponding BOLD BINs. Clades primarily found on COI and concatenated tree. ^†^ Only COI data available (from Hendrich et al. 2015).

Clade name	Localities	BOLD BIN
*Hydrobius arcticus*	Norway and Sweden	BOLD:AAC5901
*Hydrobius fuscipes rottenbergii*	Norway and Sweden	BOLD:AAC5901
*Hydrobius fuscipes fuscipes*	Norway, Sweden, Finland, Germany, Spain, Russia and Canada	BOLD:AAC5900
*Hydrobius fuscipes subrotundus*	Finland, Germany, Sweden, Norway, Italy and UK	BOLD:AAC5899
Clade I	Russia and Germany	BOLD:AAP9350
Clade II (singleton)	Portugal	BOLD:ACN8707
Clade III	Spain and Germany	BOLD:ACB2991
Clade IV	Canada	BOLD:AAH2906
Clade V	Canada and USA	BOLD:AAH0085
Clade VI	Greece ^†^	BOLD:ACO5185
Clade VII	Germany ^†^	BOLD:AAC5901

### Concatenated data (COI, H3 and ITS2 combined)

Nine monophyletic clades are found in the phylogenetic tree of the concatenated data from MrBayes (Fig. 5). All clades except the *Hydrobius fuscipes fuscipes* clade (posterior probability = 0.67) have strong support. There is strong support for Clade I and Clade II as sisters, strong support for the *Hydrobius fuscipes rottenbergii* and *Hydrobius arcticus* clades as sisters, and moderate to strong support for the relationship (Clade III, (Clade IV, (*Hydrobius arcticus*, *Hydrobius fuscipes rottenbergii*))). Specimens that were identified as different morphotypes (*Hydrobius fuscipes fuscipes* or *Hydrobius fuscipes subrotundus*) but were collected in sympatry at Rinnleiret (Nord-Trøndelag, Norway) or Motzen (Brandenburg, Germany) clustered within corresponding *Hydrobius fuscipes fuscipes* or *Hydrobius fuscipes subrotundus* clades rather than together based on locality.

### Mitochondrial COI data

Ten monophyletic groups, all of which have moderate to strong support, are found in the phylogenetic tree of COI from MrBayes (Fig. S1 in Suppl. material 3). The *Hydrobius arcticus* and *Hydrobius fuscipes rottenbergii* clades are clustered together with moderate to strong support as a single monophyletic group. There is moderate to strong support for the relationship (*Hydrobius fuscipes fuscipes*, (Clade I, Clade II)), and as in the concatenated tree (Fig. 5), strong support for Clade I and Clade II as sisters. As in the concatenated tree (Fig. 5), different morphotypes collected in sympatry cluster within the corresponding *Hydrobius fuscipes fuscipes* or *Hydrobius fuscipes subrotundus* clades rather than together based on locality (Fig. S1 in Suppl. material 3).

### Nuclear H3 data

Clade III, Clade V and *Hydrobius fuscipes subrotundus* form reciprocal monophyletic groups with moderate to strong support in the phylogenetic tree of H3 from MrBayes (Fig. S2 in Suppl. material 3). *Hydrobius arcticus*, *Hydrobius fuscipes rottenbergii* and Clade IV cluster together as a single monophyletic group with strong support, whereas Clade I, Clade II and *Hydrobius fuscipes fuscipes* are paraphyletic groups. As in the concatenated and COI trees (Fig. 5 and Fig. S1 in Suppl. material 3), different morphotypes collected in sympatry cluster with samples of the corresponding *Hydrobius fuscipes fuscipes* or *Hydrobius fuscipes subrotundus* clades rather than together based on locality (Fig. S2 in Suppl. material 3).

### Nuclear ITS2 data

Multiple reciprocally monophyletic groups are found in the phylogenetic tree of ITS2 from MrBayes (Fig. S3 in Suppl. material 3). Clade III, Clade IV and Clade V have strong support, *Hydrobius fuscipes subrotundus* has moderate support, while the *Hydrobius arcticus* clade has low to moderate support. Clade I and Clade II cluster together to form a monophyletic group with strong support. The *Hydrobius fuscipes rottenbergii* clade is a basal paraphyletic group, although all samples are identical haplotypes. The *Hydrobius fuscipes fuscipes* clade is paraphyletic, but as in the concatenated, COI and H3 trees (Fig. 5, Figs S1–S2 in Suppl. material 3), different morphotypes collected in sympatry cluster with samples of the corresponding *Hydrobius fuscipes fuscipes* or *Hydrobius fuscipes subrotundus* clades rather than together based on locality (Fig. S3 in Suppl. material 3).

### Conflict between gene trees

The three gene trees differ in the relationships between Clade I, Clade II, Clade IV and *Hydrobius fuscipes fuscipes*, *Hydrobius arcticus* and *Hydrobius fuscipes rottenbergii* (Figs S1–S3 in Suppl. material 3). Clade I and Clade II are reciprocally monophyletic groups in the COI tree, but in trees based on nuclear gene segments the two clades are either paraphyletic (H3) or their members group as a single monophyletic unit (ITS2, Fig. S3 in Suppl. material 3). The *Hydrobius fuscipes fuscipes* clade has the most variation in the COI gene segment and is paraphyletic for the nuclear gene segments (H3 and ITS2) where specimens are split in two groups. The two subgroups of *Hydrobius fuscipes fuscipes* in the H3 tree (Fig. S2 in Suppl. material 3) differ from the two subgroups in the ITS2 tree (Fig. S3 in Suppl. material 3). Clade IV, *Hydrobius arcticus* and *Hydrobius fuscipes rottenbergii* are closely related in the COI and H3 trees, while they are more basal in the ITS2 tree. Clade IV is separated genetically from the other two groups in the COI and ITS2 trees, but not in the H3 gene tree, whereas *Hydrobius arcticus* and *Hydrobius fuscipes rottenbergii* are only possible to separate genetically with ITS2 data (Fig. S3 in Suppl. material 3).

### Species delimitation analysis

#### GMYC

**Figure 6.**
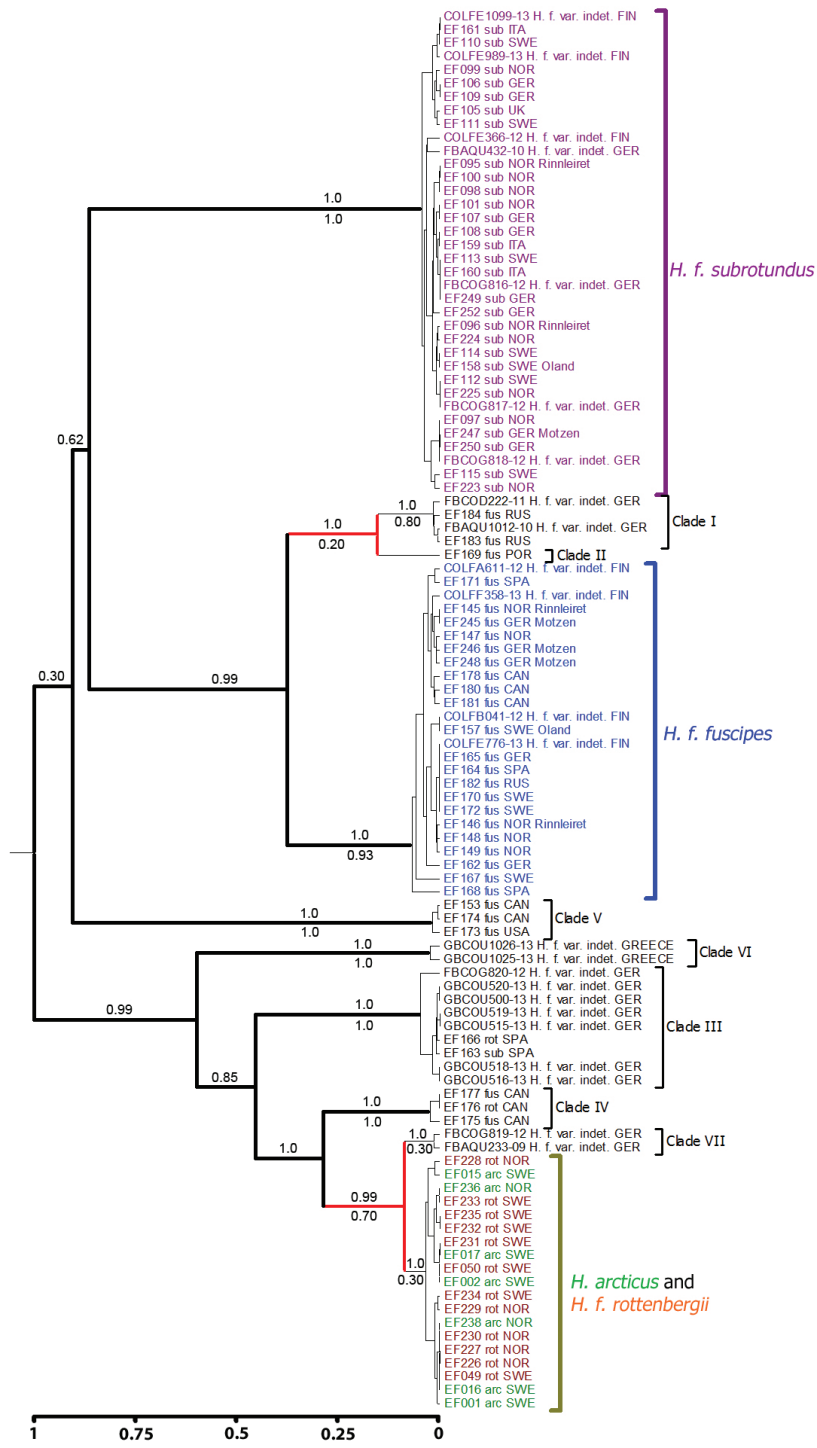
Ultrametric (strict clock) maximum clade credibility (MCC) tree used in GMYC analysis of COI. Terminal names and abbreviations as in Fig. 5. Samples from BOLD are marked with BOLD Sequence ID. Values above branches show Bayesian posterior probability support; values below branches show GMYC-support, i.e. support for the node as a GMYC-species among the alternative models of delimitation considered (95% confidence set). GMYC-support < 0.1 not shown. Splits of thick branches represent speciation events, splits of thin branches indicate within-species coalescent events and splits of red branches depend on the models considered (Table 6). Scale bar represents an artificial time scale with the root at time 1.

The ultrametric maximum clade credibility (MCC) tree from BEAST based on COI data (Fig. 6) is concordant with the non-ultrametric COI gene tree (Fig. S1 in Suppl. material 3) and supports the same clades. A GMYC model delimiting nine species with a single threshold was the maximum likelihood solution, but models delimiting eight or ten species also fall within two ΔAICc of the best GMYC model (Table 6), indicating that all three models are about equally good at explaining the data among the models compared. The log likelihood of the GMYC model at the optimal threshold (670.5) was also significantly better than the null model of a single coalescent (logL = 660.6) in a likelihood ratio test (p < 0.001). Most clades have GMYC-support values higher than 0.9 (Fig. 6), meaning that the probability of the clades being delimited as separate GMYC-species among the alternative models of delimitation (within a 95% confidence set) is higher than 0.9. Clade I and Clade II are by some models considered the same GMYC-species (GMYC-support = 0.20), but there is higher support for them being separate GMYC-species (GMYC-support = 0.80). Clade VII, *Hydrobius arcticus* and *Hydrobius fuscipes rottenbergii* are considered the same species by a majority of the models (GMYC-support = 0.70), but Clade VII is considered a separate species under some models (GMYC-support = 0.30).

**Table 6.** Model selection in GMYC. Only models within 3 Δ AICc shown. Sorted by Δ AICc. All samples are considered the same species under the null coalescent model, whereas all samples are considered separate species under the null Yule model.

Gene segment	Model	Number of clusters	Number of singletons	Log likelihood	AICc	Δ AICc	Akaike weights
COI	9 species-model	8	1	670.5	-1330.302	0.000	0.463
	10 species- model	9	1	669.7	-1328.735	1.567	0.212
	8 species-model	8	0	669.6	-1328.508	1.794	0.189
H3	Null coalescent model	1	0	504.7	-1005.209	0.000	0.174
	Null Yule model	0	84	504.5	-1004.906	0.303	0.150
ITS2	Null Yule model	0	79	465.7	-927.2162	0.000	0.172
	Null coalescent model	1	0	465.3	-926.4433	0.773	0.117
	5 species-model	5	0	468.1	-925.3851	1.831	0.0688
	9 species-model	9	0	467.7	-924.5404	2.676	0.0451

The ultrametric MCC tree from BEAST based on H3 data (Fig. S4 in Suppl. material 3) is concordant with the non-ultrametric H3 gene tree (Fig. S2 in Suppl. material 3) and supports the same clades. The GMYC model that is the maximum likelihood solution (logL = 506.1) delimited 20 species but was not significantly different from the one-species null model (lnL = 504.7) in a likelihood ratio test (p = 0.23). The model had a ΔAICc = 3.69, which is higher than both the one-species null model and the Yule null model (where all samples are different species) (Table 6), meaning that the null models are the best explanations of the data among the models compared.

**Figure 7.**
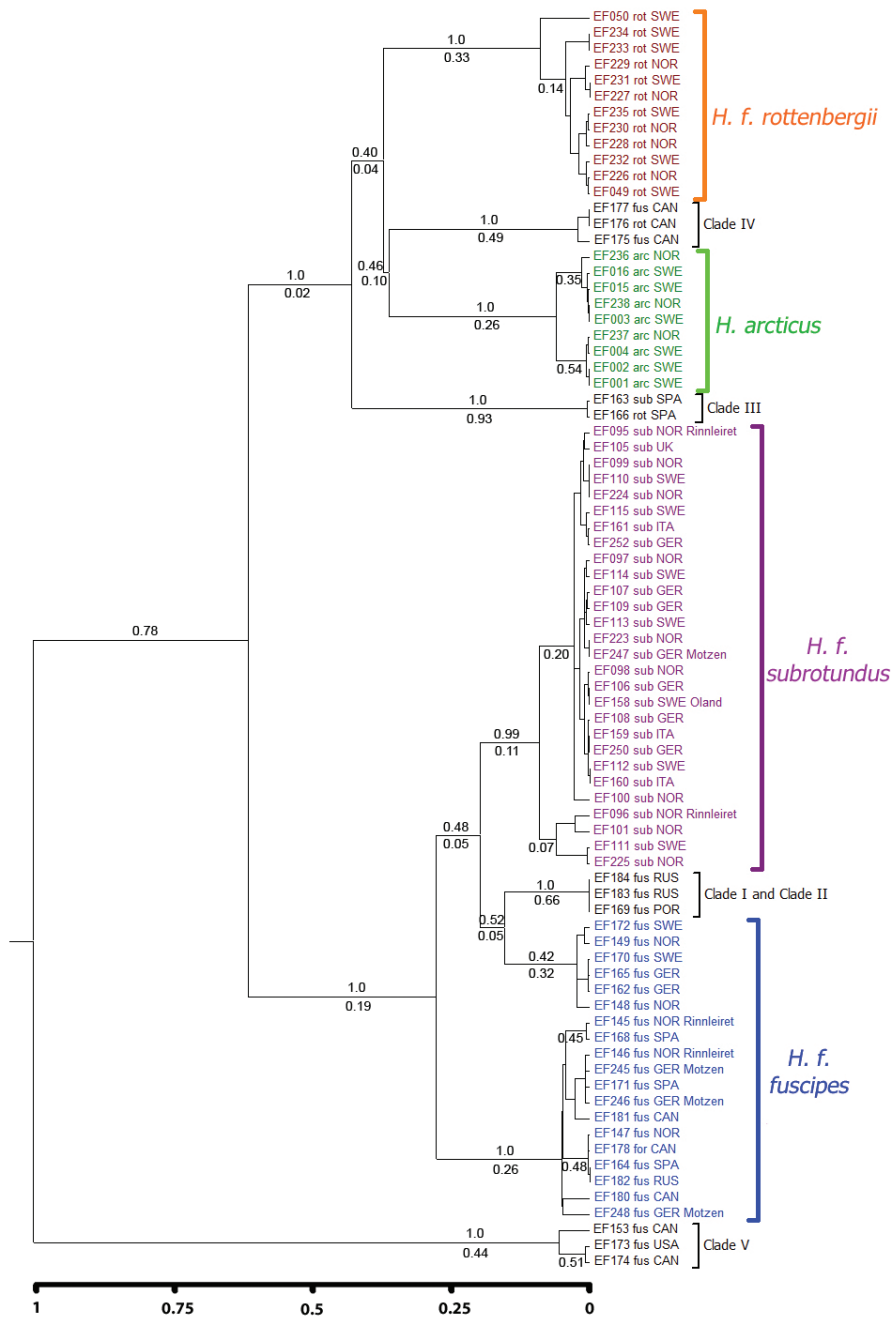
Ultrametric (strict clock) maximum clade credibility (MCC) tree used in GMYC analysis of ITS2. Terminal names and abbreviations as in Fig. 5. Values above branches show Bayesian posterior probability support (nodes with PP < 0.4 not shown); values below branches show GMYC-support. Scale bar represents an artificial time scale with the root at time 1.

The ultrametric MCC tree from BEAST based on ITS2 data (Fig. 7) is concordant with the non-ultrametric ITS2 gene tree (Fig. S3 in Suppl. material 3) and supports the same clades. A GMYC model with 5 delimited species was the maximum likelihood solution, but both the one-species null model and the Yule null model have a lower ΔAICc, whereas a 9-species model also fall within 3 ΔAICc of the best null model (Table 6). The 5-species model’s log likelihood (468.1) was not significantly different from the log likelihood of the one-species null model (465.3) in a likelihood ratio test (p = 0.061). All clades except Clade III (GMYC-support = 0.93) have low GMYC-support values (Fig. 7). There is higher support for *Hydrobius fuscipes rottenbergii*, *Hydrobius arcticus* and Clade IV being separate species (GMYC-support >0.25) than for them being the same species (GMYC-support < 0.10).

#### BPP

**Figure 8.**
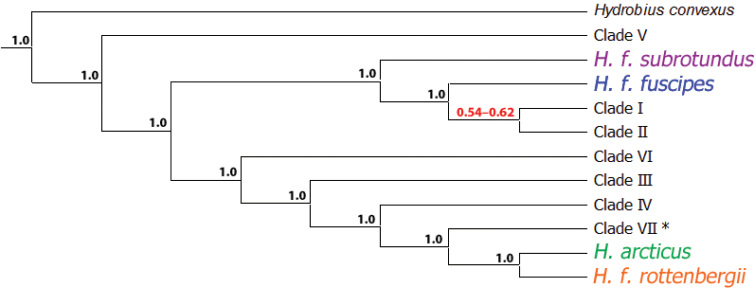
Species tree with the largest posterior probability from BPP v3.0 analyses conducted on *Hydrobius* specimens. Multi-locus data (COI, H3 and ITS2) used with *Hydrobius convexus* included as outgroup. Values above branches indicate range of split posterior probabilities, i.e. the probability for the node representing a speciation event, from four different prior-combinations. Values in red have split probabilities < 1.0. *Clade VII only delimited when specimens from Clade VII were *a priori* assigned as a potential species separate from *Hydrobius arcticus* and *Hydrobius fuscipes rottenbergii*.

BPP analyses without guide tree (BPP v3.0) were mostly conclusive and in agreement, independent of prior-combinations, parameter settings, algorithm (0 or 1), multiple runs or *a priori* sample assignments, and delimited most genetically divergent clades with posterior probabilities of 1.0 (Fig. 8 and Table 7). The largest uncertainty was whether Clade I and Clade II should be considered different species, but the posterior probability (PP) is higher for them as separate species (PP: 0.541–0.623) than for them as the same species (PP: 0.377–0.459). Clade VII was delimited as a separate species different from *Hydrobius arcticus* and/or *Hydrobius fuscipes rottenbergii* only when it was *a priori* assigned as a potential separate species. Assigning Clade VII specimens as either *Hydrobius arcticus* or *Hydrobius fuscipes rottenbergii* did not affect their posterior probability as separate species. The species trees with the highest posterior probability (Fig. 8 and Fig. S5 in Suppl. material 3) generally had similar topologies as the phylogenetic trees based on the concatenated dataset and the COI data (Fig. 5 and Fig. S1 in Suppl. material 3). Prior settings had an effect on the posterior probability of Clade I and Clade II as separate species, with a strong tendency of increasing values of tau (*τ*_0_) resulting in lower posterior probabilities and a weak tendency of increasing values of theta (*Θ*) resulting in higher posterior probabilities (Table S3 in Suppl. material 3).

**Table 7.** Posterior probabilities (PP) of delimited species from BPP v3.0, based on multi-locus data (COI, H3 and ITS2) from 111 *Hydrobius* specimens. PP range from four prior-combinations and multiple runs with different starting trees and algorithms (0 vs 1). Species delimited with PP < 0.01 are not reported. ^†^Only delimited when specimens from Clade VII were *a priori* assigned as a potential separate species from *Hydrobius arcticus* and *Hydrobius fuscipes rottenbergii*.

Delimited species	Posterior probability (range)
*Hydrobius convexus*	1.0
*Hydrobius arcticus*	1.0
*Hydrobius fuscipes rottenbergii*	1.0
*Hydrobius fuscipes fuscipes*	1.0
*Hydrobius fuscipes subrotundus*	1.0
Clade III	1.0
Clade IV	1.0
Clade V	1.0
Clade VI	1.0
Clade VII ^†^	1.0
Clade I	0.541–0.623
Clade II	0.541–0.623
Clade I and Clade II	0.377–0.459

The results from BPP v2.2 with a guide tree were very similar to the results from BPP v3.0, independent of prior-combinations, parameter settings, algorithm (0 or 1), multiple runs, guide tree topologies or *a priori* sample assignments (Fig. S6 and Table S4 in Suppl. material 3). Similar to the results from BPP v3.0, the best models delimited 11 or 12 species (including the outgroup *Hydrobius convexus*) depending on the *a priori* assignment of specimens of Clade VII. As in BPP v3.0, uncertainty was found in whether Clade I and Clade II should be considered different species, with them being separate species having a bit higher posterior probability than them being the same species. Prior settings had an effect on the split probability of Clade I and Clade II, with increased value of tau (*τ*_0_) resulting in lower split probabilities (Table S5 in Suppl. material 3). Theta (*Θ*) did not seem to affect the split probabilities.

### Morphological analyses

Only characters found to be significantly different between morphotypes are reported and discussed here. Measurements are available in Suppl. material 4.

### Genital morphometrics

**Figure 9.**
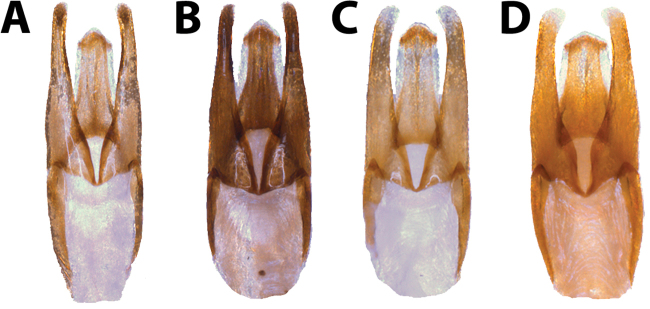
Male genitalia of *Hydrobius* morphotypes in dorsal view. **A** *Hydrobius fuscipes fuscipes* **B** *Hydrobius fuscipes subrotundus* **C** *Hydrobius fuscipes rottenbergii* **D** *Hydrobius arcticus*.

**Figure 10.**
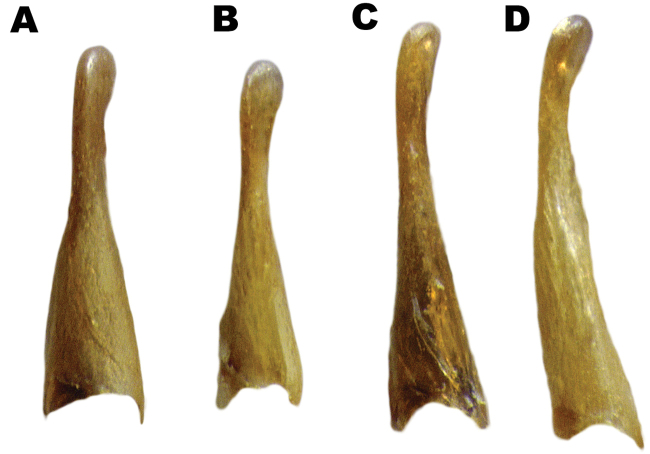
Male genitalia of *Hydrobius* morphotypes in lateral view. **A** *Hydrobius arcticus* **B** *Hydrobius fuscipes rottenbergii* **C** *Hydrobius fuscipes fuscipes* **D** *Hydrobius fuscipes subrotundus*.

Male genitalia of the *Hydrobius* morphotypes were generally similar and morphometric measurements of characters overlapped to different degrees between morphotypes (Figs 9, 10).

**Figure 11.**
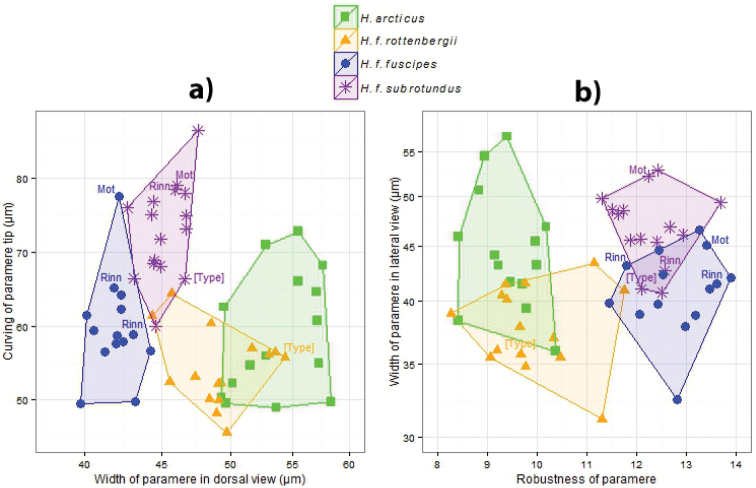
Morphometric differences between 60 (in a) and 59 (in **b**) specimens of *Hydrobius*. Two characters are plotted against each other in each figure with convex hulls used to show overlap in the data between morphotypes. Type specimens and specimens of *Hydrobius fuscipes subrotundus* and *Hydrobius fuscipes fuscipes* collected in sympatry (Rinn = locality Rinnleiret (Norway) and Mot = Motzen (Germany)) are labeled. **a** Curvature of paramere tip plotted against width of paramere in dorsal view. X-axis is in logarithmic scale **b** Width of paramere in lateral view plotted against the ratio robustness of paramere in dorsal view. Y-axis is in logarithmic scale.

Width of parameres (in logarithmic scale) in dorsal view was the most informative character and separated all morphotypes from each other, where the morphotypes explained 80.0% of the variation in the character (Table 8 and Fig. 11A). Neither body size nor an interaction between body size and morphotype were statistically significant (interaction effect: df_N_ = 3, df_D_ = 54, F = 0.0871, p = 0.967; effect of body size: df_N_ = 1, df_D_ = 57, F = 0.166, P = 0.685), meaning that body size did not affect the character. All morphotypes mean ln width of parameres were significantly different from each other, with the largest difference being *Hydrobius arcticus* having a mean that was 6.64% larger than the mean of *Hydrobius fuscipes fuscipes* (Tables S6–S7 in Suppl. material 3). The *Hydrobius fuscipes rottenbergii* type specimen had a width of paramere that is closer to the mean of the *Hydrobius arcticus* morphotype than the *Hydrobius fuscipes rottenbergii* morphotype, whereas the *Hydrobius fuscipes subrotundus* type and sympatric specimens of *Hydrobius fuscipes fuscipes* and *Hydrobius fuscipes subrotundus* had values within their respective morphotypes rather than based on locality (Fig. 11A).

**Table 8.** ANOVA/ANCOVA for effect of body size and morphotypes on different male genital characters in *Hydrobius*. Only significant effects are shown. df=degrees of freedom. ln = natural logarithm. See Material and Methods for details on character measurements.

Character (unit)	Effect	df	Mean square	F-value	p-value
Width of parameres, dorsal view (ln(µm))	Morphotype	3	0.177	79.5	< 0.001
	Residuals	58	0.00222		
Robustness of parameres	Morphotype	3	41.9	79.8	< 0.001
	Residuals	56	0.525		
Ratio between paramere length and penis length	Morphotype	3	0.0990	20.9	< 0.001
	Residuals	56	0.00474		
Width of parameres, lateral view (ln(µm))	Morphotype	3	0.122	12.6	< 0.001
	Residuals	55	0.00965		
Curvature of paramere tip (µm)	Morphotype	3	1008	22.1	< 0.001
	Residuals	56	104.5		
Length of parameres (ln(µm))	Morphotype	3	0.0534	21.9	< 0.001
	ln (body size)	1	0.0122	5.03	0.0289
	Residuals	55	0.00243		

Two characters, robustness of parameres and ratio between paramere length and penis length, separated *Hydrobius arcticus* and *Hydrobius fuscipes rottenbergii* from *Hydrobius fuscipes subrotundus* and *Hydrobius fuscipes fuscipes*. The morphotypes explained 81.1% of the variation in robustness of parameres (Table 8 and Fig. 11B). Neither body size nor an interaction between body size and morphotype were statistically significant (interaction effect: df_N_ = 3, df_D_ = 52, F = 0.395, p = 0.757; effect of body size: df_N_ = 1, df_D_ = 55, F = 1.97, p = 0.166), meaning that the character was not affected by body size. *Hydrobius arcticus* and *Hydrobius fuscipes rottenbergii* had significantly more robust parameres, represented by approximately 20–25% lower mean robustness of paramere values than *Hydrobius fuscipes fuscipes* and *Hydrobius fuscipes subrotundus* (Tables S6–S7 in Suppl. material 3). All type specimens examined and sympatric specimens of *Hydrobius fuscipes fuscipes* and *Hydrobius fuscipes subrotundus* had mean robustness values within their respective morphotypes.

**Figure 12.**
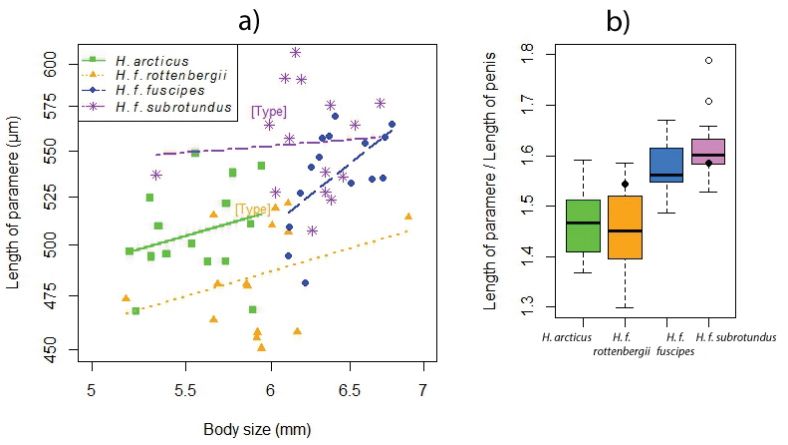
Morphometric differences between 60 specimens of *Hydrobius*. **a** Differences between morphotype and effect of body size on paramere length. Both axes are in logarithmic scale. Independently fitted lines for each morphotype are shown, slopes not significantly different. Type specimens of *Hydrobius fuscipes subrotundus* and *Hydrobius fuscipes rottenbergii* are labeled **b** Box- and whisker-plot showing differences between morphotypes on the ratio length of paramere / length of penis. Top and bottom of boxes represent first and third quartile; dark bands represent the second quartile (median); whiskers show the maximum and minimum values not including outliers (white points). Black points represent type specimens.

The morphotypes explained 52.8% of the variation in the ratio between paramere length and penis length (Table 8 and Fig. 12B). Neither body size nor an interaction between body size and morphotype were statistically significant (interaction effect: df_N_ = 3, df_D_ = 52, F = 0.1.36, p = 0.264; effect of body size: df_N_ = 1, df_D_ = 55, F = 0.145, p = 0.705). The mean of *Hydrobius arcticus* and *Hydrobius fuscipes rottenbergii* were significantly different, being approximately 7–10% lower, than the mean of *Hydrobius fuscipes fuscipes* and *Hydrobius fuscipes subrotundus* (Tables S6–S7 in Suppl. material 3). The *Hydrobius fuscipes subrotundus* type specimen had a value between the first and third quartile of its morphotype, whereas the *Hydrobius fuscipes rottenbergii* type specimen did not (Fig. 12B).

*Hydrobius arcticus* is separated from *Hydrobius fuscipes rottenbergii* and *Hydrobius fuscipes fuscipes* is separated from *Hydrobius fuscipes subrotundus* with the character width of parameres in lateral view in logarithmic scale, and the morphotypes explain 40.8% of the variation in the character (Table 8 and Fig. 11B). Neither body size nor an interaction between body size and morphotype were statistically significant (interaction effect: df_N_ = 3, df_D_ = 51, F = 0.1874, p = 0.905; effect of body size: df_N_ = 1, df_D_ = 54, F = 0.785, p = 0.380). The mean of *Hydrobius fuscipes subrotundus* was the largest and approximately 3–6% larger than the mean of *Hydrobius fuscipes rottenbergii* and *Hydrobius fuscipes fuscipes*, whereas the mean of *Hydrobius arcticus* was 4.39% larger than the mean of *Hydrobius fuscipes rottenbergii*, and these differences were significant (Tables S6–S7 in Suppl. material 3). The *Hydrobius fuscipes rottenbergii* type specimen had a value close to the mean of other *Hydrobius fuscipes rottenbergii*, whereas the type specimen of *Hydrobius fuscipes subrotundus* and sympatric specimens of *Hydrobius fuscipes fuscipes* and *Hydrobius fuscipes subrotundus* generally had somewhat overlapping values.

The *Hydrobius fuscipes subrotundus* morphotype had a significantly larger curving of the paramere tip than the other morphotypes, and the morphotypes explained 54.2% of the variation in the character (Table 8, Figs 10, 11A). Neither body size nor an interaction between body size and morphotype were statistically significant (interaction effect: df_N_ = 3, df_D_ = 52, F = 0.144, p = 0.933; effect of body size: df_N_ = 1, df_D_ = 55, F = 1.67, p = 0.202). *Hydrobius fuscipes subrotundus* mean curvature was significantly different, by being approximately 22–34% larger, than the mean of the other morphotypes (Tables S6–S7 in Suppl. material 3). The type specimens of *Hydrobius fuscipes subrotundus* and *Hydrobius fuscipes rottenbergii* were largely within their respective morphotypes, although the former had a somewhat low value. All sympatric specimens of *Hydrobius fuscipes fuscipes* and *Hydrobius fuscipes subrotundus* had values within their respective morphotypes rather than based on locality, except for a *Hydrobius fuscipes fuscipes* specimen from Motzen (Germany) which was a clear outlier (Fig. 11A).

*Hydrobius fuscipes rottenbergii* had significantly lower length of parameres than the other morphotypes, but body size did also have an effect on the character (Table 8, Fig. 12A and Table S9 in Suppl. material 3). The best model was in log-log scale and explained 56.3% of the variation in length of parameres. No statistically significant interaction was found between the morphotypes and body size (df_N_ = 3, df_D_ = 52, F = 0.842, p = 0.477), meaning that body size has the same effect on each morphotype. The common slope of the morphotypes (0.300 ± 0.134) was significantly different from zero (df = 55, t = 2.24, p = 0.0289). The intercept of *Hydrobius fuscipes rottenbergii* was significantly different, being approximately 1–2% lower, than the intercepts of the other morphotypes (Tables S8 and S9 in Suppl. material 3). This can be interpreted as *Hydrobius fuscipes rottenbergii*, on average, having significantly shorter parameres than the other morphotypes, given the same body size. The type specimen of *Hydrobius fuscipes rottenbergii* had somewhat longer parameres than what is expected for a specimen of its size, while the type specimens of *Hydrobius fuscipes subrotundus* had length of parameres close to the mean of other *Hydrobius fuscipes subrotundus* specimens of its size (Fig. 12A).

### Body characters

#### Shape of mesoventral process

**Figure 13.**
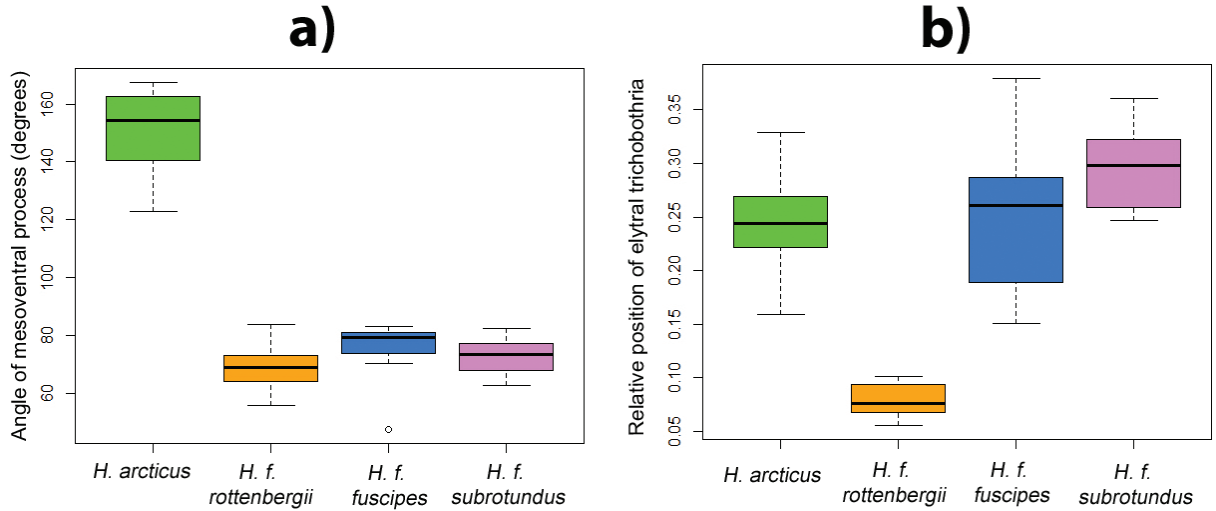
Box- and whisker-plot showing morphometric differences between morphotypes of *Hydrobius*. Top and bottom of boxes represent first and third quartile; dark bands represent the second quartile (median); whiskers show the maximum and minimum values not including outliers (white points). **a** Shape of mesoventral process. *Hydrobius arcticus* is the only morphotype with a blunt process (indicated by the higher values) **b** Relative position of trichobothria in relation to the 3rd and 5th row of elytral serial punctures. The trichobothria of *Hydrobius fuscipes rottenbergii* are positioned closer to the serial punctures than in other morphotypes (indicated by lower values).

**Figure 14.**
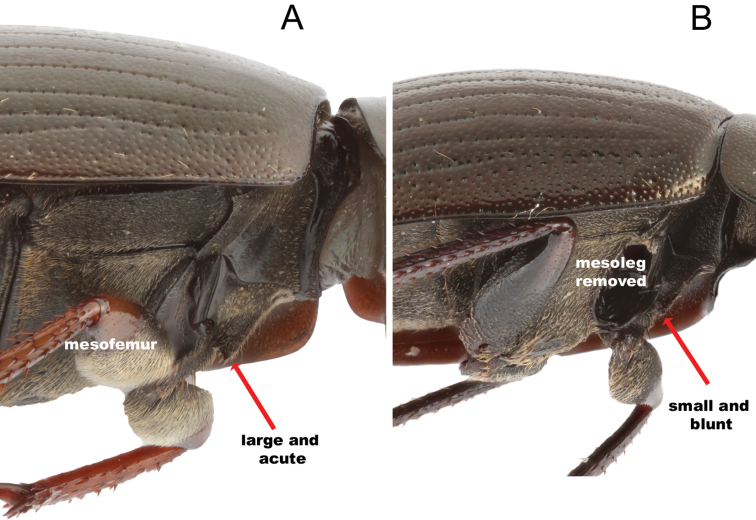
Comparison of the mesoventral process in *Hydrobius*. **A** Large and acute process found in all northern European variants of *Hydrobius fuscipes*, here represented by a specimen of *Hydrobius fuscipes fuscipes* B Small and blunt process characteristic of *Hydrobius arcticus*.

All morphotypes except *Hydrobius arcticus* had a strong or rather strong acute dentiform mesoventral process. Measurements of 10 randomly chosen specimens from each morphotype confirm this, with *Hydrobius arcticus* having higher non-overlapping values than the other morphotypes (Figs 13A, 14). The examined type specimens had the shape that is expected for their respective morphotype.

#### Relative position of trichobothria in relation to the rows of elytral serial punctures

**Figure 15.**
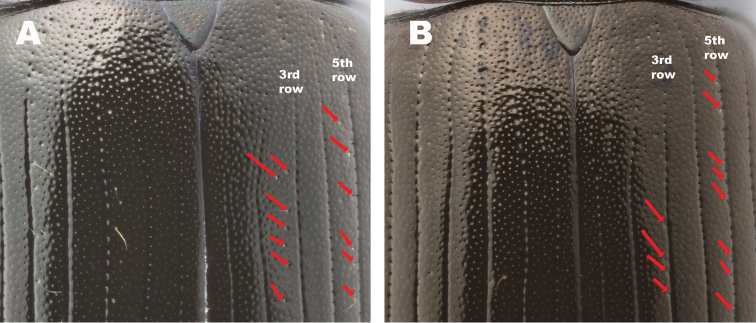
Comparison of the relative position of trichobothria (red arrows) on the elytra of *Hydrobius*. **A** Trichobothria positioned in the intervals between the 2nd and 3rd row of serial punctures, and between the 4th and 5th row. Typical positioning of trichobothria in *Hydrobius arcticus*, *Hydrobius fuscipes fuscipes* and *Hydrobius fuscipes subrotundus*, here represented by a specimen of *Hydrobius fuscipes fuscipes* **B** Trichobothria positioned in or very close to the 3rd and 5th row of serial punctures, which is characteristic of *Hydrobius fuscipes rottenbergii*.

Fennoscandian specimens of *Hydrobius fuscipes rottenbergii* had trichobothria positioned close or very close to the elytral serial punctures compared to the other morphotypes that had trichobothria located further into the elytral intervals (Fig. 15). This was only consistent for trichobothria located anteriorly on the elytra posterior to the scutellum. Trichobothria located laterally to the scutellum were generally close to the elytral serial punctures for all morphotypes, whereas trichobothria located on the posterior half of the elytra tended to be positioned further into the elytral intervals in all morphotypes. Some trichobothria deviated in relative position within the specimens, but an average of the position of several trichobothria was consistent for the morphotypes. Initial measurements of the position of trichobothria within the appropriate area showed that the average position of the Fennoscandian specimens of *Hydrobius fuscipes rottenbergii* were non-overlapping with the other morphotypes (Fig. 13B), thus the relative position was not measured more thoroughly. This pattern was not as apparent for specimens collected outside of Fennoscandia, where some specimens identified as the *Hydrobius fuscipes rottenbergii* morphotype had a relatively larger proportion of trichobothria located in the intervals than the Fennoscandian *Hydrobius fuscipes rottenbergii* specimens. The examined type specimens had trichobothria located as expected for their respective morphotype.

#### Color of legs

On average *Hydrobius fuscipes subrotundus* had darker femora and tibiae than the other morphotypes, but some overlap was found between the color of *Hydrobius fuscipes subrotundus* and *Hydrobius fuscipes fuscipes*. Color differences were more consistent for the femora than for the tibiae, although color of the femora often became lighter towards the trochanter. Specimens with entirely dark legs were always of the *Hydrobius fuscipes subrotundus* morphotype, but overlap was found when comparing *Hydrobius fuscipes subrotundus* specimens with less dark legs with the *Hydrobius fuscipes fuscipes* specimens with the darkest legs. On the other hand, entirely yellow legs are common in *Hydrobius fuscipes fuscipes*, but are never found in *Hydrobius fuscipes subrotundus*. Specimens of *Hydrobius fuscipes subrotundus* collected in sympatry with specimens of *Hydrobius fuscipes fuscipes* had darker legs than the *Hydrobius fuscipes fuscipes* specimens. The type specimen of *Hydrobius fuscipes subrotundus* had dark legs, whereas type specimens of other morphotypes had lighter legs.

#### Body shape (Elytral Index)

**Figure 16.**
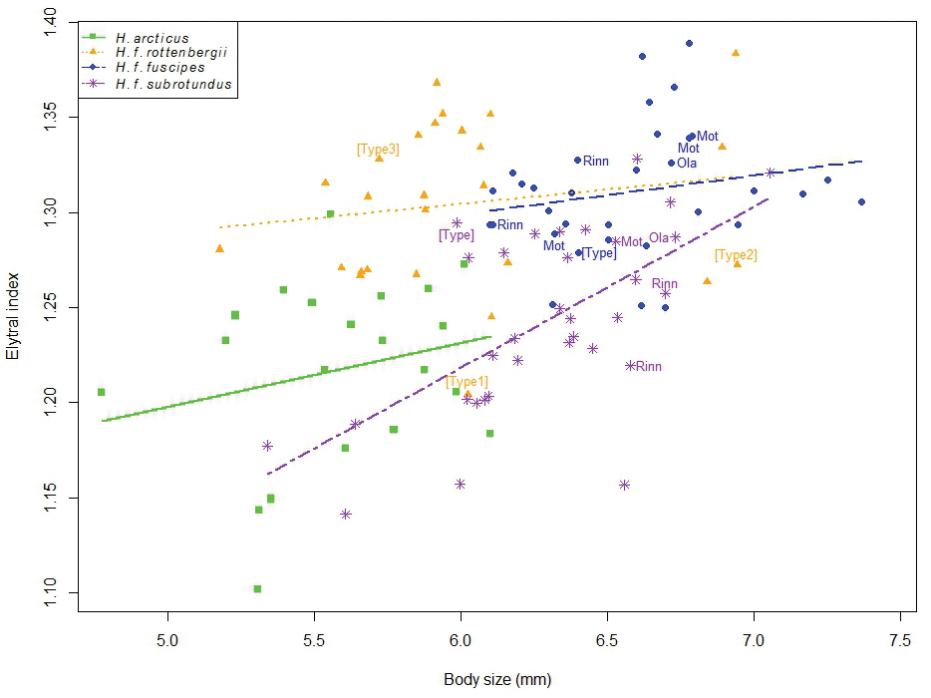
Morphometric differences between morphotypes and effect of body size on Elytral Index (EI) of *Hydrobius*. EI = length of the elytra / maximum width of elytra. 113 specimens measured. Independently fitted lines for each morphotype are shown, slopes not significantly different. Type specimens and specimens of *Hydrobius fuscipes subrotundus* and *Hydrobius fuscipes fuscipes* collected in sympatry (Rinn = locality Rinnleiret (Norway), Mot = Motzen (Germany) and Ola = Öland (Sweden)) are labeled.

Both morphotypes and body size had a significant effect on the Elytral Index (EI = length of the elytra / maximum width of elytra), with the best model explaining 51.0% of the variance in EI (Table 9 and Fig. 16). No statistically significant interaction was found between the morphotypes and body size (df_N_ = 3, df_D_ = 105, F = 2.56, p = 0.0591), meaning that body size affect each morphotype in the same way (i.e. the morphotypes have a common slope). The common slope (0.0381 ± 0.0107) was significantly different from zero (df = 108, t = 3.55, p < 0.001) and means that EI increases by 0.0381 for each mm increase in body size in all morphotypes.

**Table 9.** ANCOVA for effect of body size and morphotypes on Elytral Index (EI) in *Hydrobius*. Only significant effects are shown. df=degrees of freedom. See Material and Methods for details on character measurements.

Effect	df	Mean square	F-value	p-value
Morphotype	3	0.0558	33.188	< 0.001
Body size	1	0.0212	12.615	< 0.001
Residuals	108	0.00168		

**Figure 17.**
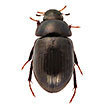
Habitus of *Hydrobius* morphotypes in dorsal view. **A** *Hydrobius arcticus* **B** *Hydrobius fuscipes rottenbergii* **C** *Hydrobius fuscipes fuscipes* **D** *Hydrobius fuscipes subrotundus*.

The intercepts of *Hydrobius fuscipes subrotundus* and *Hydrobius arcticus* were significantly different, being approximately 5–7% lower, than the intercepts of *Hydrobius fuscipes fuscipes* and *Hydrobius fuscipes rottenbergii* (Tables S8 and S10 in Suppl. material 3). This can be interpreted as *Hydrobius arcticus* and *Hydrobius fuscipes subrotundus* having on average an EI value that is 5–7% lower than the values of *Hydrobius fuscipes fuscipes* and *Hydrobius fuscipes rottenbergii*, given that the individuals being compared have identical body size. This means that *Hydrobius arcticus* and *Hydrobius fuscipes subrotundus* generally have a more convex body than *Hydrobius fuscipes fuscipes* and *Hydrobius fuscipes rottenbergii* (Fig. 17). Sympatric specimens of *Hydrobius fuscipes fuscipes* and *Hydrobius fuscipes subrotundus* generally had EI-values within their respective morphotypes rather than based on locality (Fig. 16). The type specimen of *Hydrobius fuscipes subrotundus* had an EI value above what is expected for a specimen of its size, while one of the type specimens of *Hydrobius fuscipes rottenbergii* had an EI value below most *Hydrobius fuscipes rottenbergii* specimens measured (Fig. 16). The type specimen of *Hydrobius fuscipes fuscipes* had an EI-value close to what is expected for a specimen of its size, although somewhat low.

## Discussion

### Phylogenetic relationships

The nuclear gene segments H3 and ITS2 had comparatively low genetic variation (Table 4) and results based on these are therefore sensitive to editing and sequencing errors. However, subsamples of all markers were sequenced twice with the same result and all troublesome sequences were checked multiple times to eliminate the effect of wrong base calls. The low variation in the nuclear gene segments may have resulted in overparameterising of the phylogenetic models and explain why some expected clades in the H3 and ITS2 trees are basal paraphyletic groups without a common node (e.g. *Hydrobius fuscipes rottenbergii* specimens with identical haplotypes in ITS2, Fig. S3 in Suppl. material 3). Since COI data are the most variable, the concatenated dataset and corresponding tree (Fig. 5) is highly affected by the COI data. However, Clade III, Clade V and the *Hydrobius fuscipes subrotundus* clade were supported as reciprocal monophyletic groups by all markers, suggesting that there is informative data in the nuclear gene segments.

The ITS2 results differ from the other gene trees by the placement of *Hydrobius fuscipes rottenbergii*, *Hydrobius arcticus* and Clade IV basally in the tree (Fig. S3 in Suppl. material 3). This is possibly due to the outgroup *Hydrobius convexus* having very divergent ITS2 sequences (Table 4, Fig. S3 in Suppl. material 3) and that the substitution model best fit for the ingroup was unfit to use on the complete dataset (Table S2 in Suppl. material 3). As a strict clock model was preferred in the Bayes factor test using stepping stone sampling, the root inferred in the ultrametric tree (Fig. 7) is more appropriate than the root inferred by outgroup comparison under a non-clock model.

Interestingly, a more complex partition scheme and substitution model was also found for the H3 dataset when including as opposed to excluding the outgroup (Table S2 in Suppl. material 3), suggesting that *Hydrobius convexus* is quite distantly related to *Hydrobius fuscipes* and *Hydrobius arcticus*. This is supported by Short and Liebherr (2007) and Short and Fikáček (2013) who suggested that *Hydrobius* is paraphyletic with respect to species of *Limnocyclus*, *Limnoxenus*, *Hydramara, Sperchopsis, Ametor* and *Hybogralius*. Short and Fikáček (2013) used molecular data and found evidence for *Hydrobius fuscipes* being more closely related to species of *Ametor* and *Sperchopsis* than to *Hydrobius melaenus*. However, it is not clear which genetic groups of *Hydrobius fuscipes* they had sampled and they did not include *Hydrobius convexus* in their study. While *Hydrobius convexus* may not be the ideal outgroup for phylogenetic studies of the *Hydrobius fuscipes* species complex, it was the only other species of *Hydrobius* available to us for this study.

The most likely general explanation for the conflicting phylogenetic patterns in the gene trees (Figs S1–S3 in Suppl. material 3) is limited variation in the nuclear gene segments and incomplete lineage sorting (Pamilo and Nei 1988). Lack of variation is the best explanation for members of Clade IV being identical to *Hydrobius arcticus* and *Hydrobius fuscipes rottenbergii* in the H3 gene tree (Fig. S2 in Suppl. material 3) and it is a likely explanation for Clade I and Clade II not being divergent in the H3 and ITS2 trees. Incomplete lineage sorting is probably also the best explanation for *Hydrobius fuscipes fuscipes* being paraphyletic in the nuclear trees. The *Hydrobius fuscipes fuscipes* group is the most divergent group in the COI tree (Fig. S1 in Suppl. material 3) and it would be interesting to see if more variable nuclear markers would group the specimens together in nuclear gene trees.

The most interesting conflict between the gene trees was in the lack of reciprocal monophyly of *Hydrobius arcticus* and *Hydrobius fuscipes rottenbergii* for COI and H3. Specimens belonging to these morphotypes grouped together with almost identical sequences in both the COI and H3 gene trees (Figs S1 and S2), but were placed in moderately supported separate monophyletic groups in the ITS2 gene tree and the tree based on the concatenated dataset (Fig. 5 and Fig. S3 in Suppl. material 3). The H3 data probably did not separate the morphotypes due to low variation in this marker. However, this explanation is unlikely for the more variable COI marker. A possible explanation is introgression due to rare hybridization events between the morphotypes after geographical separation. Selective sweeps, where mtDNA was affected more strongly than nDNA, could lead to the observed pattern if one of the ancestral morphotypes had parts of mtDNA that led to higher fitness in the hybrid (Ballard and Whitlock 2004). It could be interesting to test for selective sweeps through for example linkage equilibrium tests, but this would require genetic data with more variation appropriate for population genetic studies.

Despite having widely different habitats and not being known to occur in sympatry, the *Hydrobius arcticus* and *Hydrobius fuscipes rottenbergii* morphotypes may have had a relatively recent hybridization event resulting in very similar COI sequences. A possible explanation for when this event occurred, although speculative, is related to their habitats at the end of the last ice age (10–14 000 years ago). As the ice cover melted, what was then coastal areas close to the retracting ice had similar environmental conditions as alpine/arctic areas do today (Lokrantz and Sohlenius 2006). As a result, the two morphotypes could have been sympatric at the time and may have hybridized at low frequency resulting in mixing of mtDNA.

### Genetic species delimitation

GMYC results based on COI data (Fig. 6 and Table 6) strongly support most of the genetically divergent clades as distinct species, the largest uncertainty being whether Clade I and Clade II are separate species and whether Clade VII is the same species as *Hydrobius arcticus* and *Hydrobius fuscipes rottenbergii*. GMYC assumes complete lineage sorting, no hybridization and species monophyly in the gene tree (Pons et al. 2006), requirements that might be violated in the COI data. The specimens within Clade VII were from Hendrich et al. (2015) and identified as *Hydrobius fuscipes* var. indet, making it difficult to know which morphotype they morphologically resemble. These specimens’ geographic localities are inland Germany (Bavaria), which does not fit either *Hydrobius fuscipes rottenbergii* (coastal localities) or *Hydrobius arcticus* (alpine-arctic localities). Morphology and ITS2 gene sequences from these specimens might reveal if Clade VII is a valid species.

The GMYC analyses on the nuclear gene segments were less informative, most likely because of the low variation. This appears to be especially true for the H3 data which was best explained by the null models (Table 6). The ITS2 data had four models within 3 Δ AICc (Table 6), with both null models having the lowest Δ AICc, which means that all models are about equally good at explaining the data among the models compared (Burnham and Anderson 2002). The GMYC-support values (in Fig. 7) are more interesting for the ITS2 data, as they suggest that Clade IV, *Hydrobius fuscipes rottenbergii* and *Hydrobius arcticus* should be delimited as separate species (GMYC-support 0.26–0.49). However, GMYC can be prone to overdelimitation (Carstens et al. 2013) which may also explain this pattern. The fact that our *Hydrobius arcticus* specimens have relatively high support (GMYC-support 0.36–0.54) for being split into two separate species despite having identical haplotypes illustrates this well. The *Hydrobius fuscipes fuscipes* morphotype, which is paraphyletic in the ITS2 gene tree, is delimited as several species probably because the GMYC method assumes species monophyly in the gene tree (Pons et al. 2006).

The BPP results, both with version 2.2 and v3.0 (Table 7, Fig. 8, Table S4 and Fig. S6 in Suppl. material 3) strongly support most of the genetically divergent clades that were reliably delimited with GMYC as separate species, suggesting that the genetic differences found between the clades are significant enough to consider the groups different species. Clade I and Clade II were the clades with the lowest split probabilities overall and since the priors were shown to have an effect on the split probabilities (Table S3 and S5 in Suppl. material 3), these specimens may be of the same species. However, Clade II is only one specimen, meaning that the statistical power of the BPP analyses is low when testing the delimitation of Clades I and II. More specimens from these groups would be required to arrive at a more reliable BPP result.

The BPP results delimited Clade VII, *Hydrobius arcticus* and *Hydrobius fuscipes rottenbergii* as separate species in all analyses, strongly suggesting that they are different species (Table 7, Fig. 8 and Fig. S6C–E in Suppl. material 3). Interestingly, *a priori* assigning specimens of Clade VII as *Hydrobius arcticus* or *Hydrobius fuscipes rottenbergii* did not affect the split probability of the two morphotypes (Table 7 and Fig. S6B in Suppl. material 3), showing the importance of the *a priori* assignment of samples. The fact that Clade VII consisted of only two specimens (with only COI data available) may explain why it did not affect the split probability of *Hydrobius arcticus* and *Hydrobius fuscipes rottenbergii*.

Results from BPP v3.0 were very similar to the results from BPP v2.2, probably because the species trees with highest posterior probabilities in BPP v3.0 were very similar to the guide trees used in BPP v2.2 analyses (Figs S5 and S6 in Suppl. material 3). The guide tree and the number of terminals (i.e. potential species) can affect the results of the BPP analyses (Leache and Fujita 2010), but both BPP versions gave similar results, indicating that the guide trees used in BPP v2.2 likely did not affect the results. Conceptually, however, the new version of BPP represents a great step forward. It brings multi-locus Bayesian species delimitation under the multispecies coalescent (MSC) model into the realm of discovery methods at least for small datasets (although apart from computational limitations the presently implemented priors may be inappropriate, see Yang and Rannala 2014). Even when not fully a discovery method, minimum population level assignments may often be straightforward and BPP version 3 will jointly infer species delimitation and species phylogeny under the MSC while taking gene tree uncertainties (topology and branch lengths) into account.

Overall, both GMYC and BPP suggest that Clades III, IV, V, VI, VII, *Hydrobius arcticus*, *Hydrobius fuscipes fuscipes*, *Hydrobius fuscipes rottenbergii* and *Hydrobius fuscipes subrotundus* are sufficiently genetically divergent to be considered separate species, whereas they do not agree upon whether or not Clades I and II are the same species. BPP uses multi-locus data, is not affected by incomplete lineage sorting and can handle small amounts of hybridization between species (Zhang et al. 2011). This may explain why it provides a clearer result in terms of species boundaries than the GMYC method for our data.

### Male genital morphometrics

Several significant differences in genital characters were found between the morphotypes (Table S6 and S9 in Suppl. material 3). The effect size (i.e. how large the differences are) do vary among the characters, with three of the characters (length of parameres and width of parameres in both lateral and dorsal view) having a relatively low effect size of less than 7% difference at most. With such a relatively low effect size, it is difficult to observe the difference without doing measurements, whereas the robustness of parameres had an effect size of approximately 20% and this difference is observable in Fig. 9. *Hydrobius arcticus* and *Hydrobius fuscipes rottenbergii* clearly have more robust parameres than *Hydrobius fuscipes fuscipes* and *Hydrobius fuscipes subrotundus*. Similarly, the effect size in the character curvature of the paramere tip is approximately 25% and is also observable in Fig. 10. In this case the *Hydrobius fuscipes subrotundus* morphotype (Fig. 10D) had a clearly more strongly curved paramere tip than the other morphotypes (Fig. 10A–C).

Some overlap was found between at least two of the morphotypes in all characters (Figs 11 and 12), which may suggest that some hybridization occur between the morphotypes. However, this is not concordant with the genetic data, as one would expect different morphotypes to group together to a larger degree in the phylogenetic trees. Any substantial hybridization would likely also have led to the morphotypes not being delimited in BPP. If hybridization does occur it is likely that other post-mating isolation mechanisms may be at work, for example infertile hybrids.

Several of the genital characters examined here are correlated to each other, meaning that the number of independent characters examined is low. For instance, the robustness of parameres is a ratio between the character length of parameres and the character width of paramere in dorsal view. These correlations also make it probable that an outlier in one character will also be an outlier in another character. The relatively low number of specimens measured (approximately 15 of each morphotype, limited by the number of sequenced specimens) make the results more prone to artifacts. However, several of the differences were highly statistically significant (p < 0.001), suggesting that coincidence is not a likely explanation.

*Hydrobius fuscipes subrotundus* and *Hydrobius fuscipes fuscipes* specimens were collected in sympatry, but grouped nevertheless with specimens of their respective morphotype in all phylogenetic trees (Fig. 5 and Figs S1–S3 in Suppl. material 3). The genital morphometric analyses also moderately support this observation and the sympatric specimens of different morphotypes have no overlap in width of parameres in dorsal view and very little overlap in curvature of paramere tip (Fig. 11A). The low number of morphologically compared sympatric specimens (n = 5) makes this comparison inconclusive. However, in concert with the genetic data, genital morphology does indicate that these belong to separate species.

### Diagnostic body characters

*Hydrobius arcticus* is the morphotype easiest to identify, while *Hydrobius fuscipes fuscipes* can be difficult to separate from *Hydrobius fuscipes subrotundus*. The latter may have led to misidentifications of specimens, especially for specimens outside of northern Europe. Our genetic data indicate the presence of six or seven additional species outside of northern Europe. To enable reliable morphological identification of these, more specimens from a larger geographical range should be analyzed, especially if they are to be described as species new to science.

The relative position of trichobothria is one of the characters Hansen (1987) used to separate *Hydrobius fuscipes rottenbergii* from the other morphotypes, but our results show that the character is only useful under certain conditions (only consistent for trichobothria located on the upper part of the elytra posterior to the scutellum) and only works on Fennoscandian morphotypes. This is also evident from Fig. 5, where 2 specimens identified as *Hydrobius fuscipes rottenbergii* from outside northern Europe belong to the genetically divergent Clade III and Clade V.

Body shape (EI) has been used to separate *Hydrobius fuscipes fuscipes* from *Hydrobius fuscipes subrotundus* (Hansen (1987), but is not ideal as a diagnostic character since body size affects the character and there is some overlap between the morphotypes (Fig. 16 and Table 9). The best use of the character is when comparing specimens of similar size. A combination of body shape, color of legs, and the male genital character curvature of the paramere tip may be the best way to separate *Hydrobius fuscipes fuscipes* from *Hydrobius fuscipes subrotundus*, preferably comparing them side by side. If the specimen has an extreme character value (e.g. entirely black or yellow femora or a very clearly short and convex body shape) it may not be necessary to look at all the characters.

The large number of listed synonyms for each species (especially *Hydrobius fuscipes*) makes certain association of morphotypes with nominal species challenging. Type specimens of senior synonyms were examined when available, but we were unable to borrow types of *Hydrobius arcticus*, and could not study the genitalia of the *Hydrobius fuscipes fuscipes* type. The position of trichobothria, shape of mesoventral process and color of legs were as expected for type specimens, suggesting that the correct name have been applied to the different morphotypes analyzed. However, other quantitative measurements of the types were not necessarily concordant with measurements from other specimens of the respective morphotypes. The type of *Hydrobius fuscipes fuscipes* generally grouped together with other *Hydrobius fuscipes fuscipes* specimens, but the *Hydrobius fuscipes subrotundus* type and some of the *Hydrobius fuscipes rottenbergii* types had character values, both on body and genitalia, that were larger or smaller than most of their respective morphotypes (e.g. Fig. 12). These incongruities indicate that wrong names may have been applied or that the few type specimens examined represent outliers for certain measurements. Correlation between some of the characters, especially ratios that use elytra or paramere lengths, can also explain why a specimen is an outlier in more than one character.

The large number of synonyms must be considered when dealing with the genetically divergent clades (Table 5) as potential separate and valid new species, complicating the taxonomic work in *Hydrobius*. *Hydrobius arcticus* has been reported from northeastern Algeria (İncekara 2007), Iran (Ghahari and Jedryczkowski 2011) and Turkey (Mart et al. 2006). Our results suggest that these specimens may have been wrongly identified, as there are several potential cryptic species within *Hydrobius*. It is not unreasonable to think that *Hydrobius arcticus* specimens from the Mediterranean region and the Middle-East actually are something different from the northern European artic/alpine *Hydrobius arcticus*. Mart et al. (2006) provided a sketch of the male genitalia of their *Hydrobius arcticus*. Assuming that the sketch is accurate, the paramere robustness ratio is 13–15, too high to be *Hydrobius arcticus*.

COI barcodes could not distinguish *Hydrobius fuscipes rottenbergii* from *Hydrobius arcticus*, making this an example of where DNA barcoding fails to identify different morphospecies. Using ITS2 as an additional marker will separate these species, however. On the other hand, traditional DNA barcodes can be used to separate all other genetically divergent clades (Fig. S1 in Suppl. material 3), including potentially cryptic species within the *Hydrobius fuscipes* complex. Data in BOLD currently identified as *Hydrobius fuscipes* need to be revised to reflect this for the database to be efficient in the identification of closely related species in the *Hydrobius fuscipes* complex.

This study shows that using multiple methods, based on both morphology and molecular data, is important in species delimitation studies. This has also been shown in other integrative taxonomic studies, where using only one method to delimit species can and often will result in erroneous delimitations (e.g. Carstens et al. 2013; Padial et al. 2010; Schlick-Steiner et al. 2010).

Overall our results correspond well with the conclusion of Lindberg (1943) on the variants of *Hydrobius fuscipes* being different species. Compared to Lindberg (1943), this study expands the taxon sampling by including *Hydrobius arcticus*, a close relative to *Hydrobius fuscipes rottenbergii* and by looking at genital morphometrics and genetic data in addition to traditional diagnostic characters. We also show that populations from central and southern Europe and North America might be additional species in the *Hydrobius fuscipes* species complex.

## Conclusions, taxonomy and key

The four *Hydrobius* morphotypes examined in northern Europe should be regarded as separate species and elevated:

*Hydrobius arcticus* Kuwert, 1890

*Hydrobius fuscipes* (Linnaeus, 1758)

*Hydrobius rottenbergii* Gerhardt, 1872, **stat. n.**

*Hydrobius subrotundus* Stephens, 1829, **stat. n.**

The fact that *Hydrobius rottenbergii* is much more closely related to *Hydrobius arcticus* (based on both genetic data and similarity in male genitalia), the morphotype of which has been regarded as a separate and valid species for the longest time, than to the other *Hydrobius fuscipes* variants clearly indicates that it is a valid species. The consistent difference in the position of trichobothria in the elytral serial punctures rather than in the elytral intervals as in *Hydrobius fuscipes* and *Hydrobius subrotundus*, is further evidence of significant morphological divergence that cannot be disregarded as intraspecific variation since it covaries with 1) the male genitalia of short and broad *arcticus*-type, 2) the genetic evidence, and 3) the difference in ecological niche being coastal rock pools.

The strongest argument for *Hydrobius subrotundus* being a separate species is the fact that despite being sympatric with *Hydrobius fuscipes*, they are well differentiated clades genetically which covaries with significantly different, albeit overlapping, genitalic and body shape characters as well as partly subdivided ecological niches. This indicates little or no gene flow between the species despite living in sympatry, which rules out treating them as subspecies according to the most commonly used concept (Mayr and Ashlock 1991). We are aware that the taxonomic level of subspecies is sometimes used in another sense, sometimes as a kind of compromise bin for complex or uncertain situations. However, we feel following a precise and predictive (hence testable) definition for subspecies is preferable for scientific progress. Even though our study is limited by focusing on a subset of the complete geographical range of the *Hydrobius fuscipes* complex, our data is clear enough to reject both a conspecific and subspecific status of the four examined taxa in northern Europe.

There is a chance that the names *Hydrobius subrotundus* and *Hydrobius rottenbergii* are inappropriately used for the clades here referred to (genetic data were not retrieved from type material). Type localities are in England for *Hydrobius subrotundus* and in Central Europe for *Hydrobius rottenbergii* and we have shown that specimens that could be associated with these names from Central Europe may represent additional species, genetically distinct. To solve the situation in central as well as southern Europe will require further taxonomic work for sure. However, we consider recognition of four clearly valid species in northern Europe under traditional names the best stimulus for further decrypting the *Hydrobius fuscipes* complex in the rest of Europe, east Palearctic and the Nearctic. In fact, since *Hydrobius fuscipes* has for a long time been suspected or even known to be a species complex by Hydrophilid-workers, yet still not solved or moved further to a solution, indicates that it is a multifaceted problem that may need to be solved step by step. Future studies benefit from the possibility of sequencing DNA fragments from old type material and in this way match type specimens with appropriate genetic groups and will show if alternative names should be applied. These comparisons in combination with conducting morphological analyses of the genetically divergent clades not present in northern Europe (this study; Hendrich et al. 2015) should yield conclusive results regarding the taxonomy of these potential additional species. One should keep in mind, however, that deep genetic divergence in itself does not necessarily prove heterospecific status of individuals. Deep divergences may result from the survival of two or more old and divergent copies at a genetic locus within a lineage with full panmixis. Covariation with differences in other characters is a prerequisite to reject this situation as panmixis is predicted to erase any population divergence in other traits.

### Identification key to *Hydrobius* species of northern Europe

**Table:** 

1	Mesoventral process blunt, angle >100° (Fig. 14B). Body size smaller (length of pronotum + elytra = 4.6–6.2 mm). Male parameres robust (Fig. 9D). Alpine-arctic environment.	***Hydrobius arcticus***
–	Mesoventral process acute and dentiform, angle <100° (Fig. 14A). Body size larger (length of pronotum + elytra = 5.1–7.4 mm). Male parameres more elongate and thin (Fig. 9A–B), or if robust (Fig. 9C) then trichobothria on anterior half of elytra situated in, or very close to, the 3th and 5th row of elytral serial punctures (Fig. 15B).	**2**
2	Trichobothria on anterior half of elytra situated in, or very close to, the 3rd and 5th row of elytral serial punctures (Fig. 15B). Male parameres robust (Fig. 9C). Coastal rock pools.	***Hydrobius rottenbergii***
–	Trichobothria on anterior half of elytra situated in the intervals between the 2nd and 3rd, and between the 4th and 5th row of serial punctures (Fig. 15A). Male parameres elongate and thin (Figs 9A–B).	**3**
3	Body shape generally compact and shorter (Elytral length/width = 1.14–1.33, Fig. 17D). Male parameres in lateral view significantly curved towards apex (Fig. 10D). Legs dark brown to black. More shaded or colder waters and at vegetation rich edges of slow flowing waters.	***Hydrobius subrotundus***
–	Body shape generally more elongate (Elytral length/width = 1.25–1.40, Fig. 17C). Male parameres in lateral view weakly curved towards apex (Fig. 10C). Legs yellow to dark brown. Characteristic species in open sun exposed, temporary or eutrophic, stagnant pools and ponds.	***Hydrobius fuscipes***

## Supplementary materials

Supplementary material 1 Specimen information Data type: specimen data Explanation note: Excel file containing information (locality data, voucher info, gender, BOLD ID) about all specimens that where measured and/or sequenced. This dataset is made available under the Open Database License (http://opendatacommons.org/licenses/odbl/1.0/). The Open Database License (ODbL) is a license agreement intended to allow users to freely share, modify, and use this Dataset while maintaining this same freedom for others, provided that the original source and author(s) are credited.Erlend I. Fossen, Torbjørn Ekrem, Anders N. Nilsson, Johannes Bergsten

Supplementary material 2 Additional morphological characters Data type: Word file Explanation note: Word file containing a list and descriptions of additional morphological characters that were measured. Contains 11 external body and 8 male genital characters. This dataset is made available under the Open Database License (http://opendatacommons.org/licenses/odbl/1.0/). The Open Database License (ODbL) is a license agreement intended to allow users to freely share, modify, and use this Dataset while maintaining this same freedom for others, provided that the original source and author(s) are credited.Erlend I. Fossen, Torbjørn Ekrem, Anders N. Nilsson, Johannes Bergsten

Supplementary material 3 Supplementary tables and figures Data type: Tables and figures Explanation note: Additional tables (S1-S10) and figures (S1-S6). Phylogenetic trees, BPP results, post-hoc comparisons of morphological characters etc. This dataset is made available under the Open Database License (http://opendatacommons.org/licenses/odbl/1.0/). The Open Database License (ODbL) is a license agreement intended to allow users to freely share, modify, and use this Dataset while maintaining this same freedom for others, provided that the original source and author(s) are credited.Erlend I. Fossen, Torbjørn Ekrem, Anders N. Nilsson, Johannes Bergsten

Supplementary material 4 Morphological dataset Data type: Morphological measurements Explanation note: Excel file containing the complete morphological measurements. Includes a second data sheet with non-abbreviated variables and units for the measurements. This dataset is made available under the Open Database License (http://opendatacommons.org/licenses/odbl/1.0/). The Open Database License (ODbL) is a license agreement intended to allow users to freely share, modify, and use this Dataset while maintaining this same freedom for others, provided that the original source and author(s) are credited.Erlend I. Fossen, Torbjørn Ekrem, Anders N. Nilsson, Johannes Bergsten
