# Supplementary material for: Species delimitation in northern European water scavenger beetles of the genus Hydrobius (Coleoptera, Hydrophilidae)
Source: Zookeys. 2016 Feb 16;(564):71–120. doi: 10.3897/zookeys.564.6558 (PMC4820092; doi:10.3897/zookeys.564.6558)
Supplement: Supplementary material 2 — Additional morphological characters [file zookeys-564-071-s002.docx]

## Supplementary Information 2: additional morphological characters

**Body characters**:

1. Body length: measured from the front margin of the labrum to the elytral apex.
2. Maximum length of abdomen.
3. Maximum width of abdomen.
4. Maximum length of proventrite.
5. Maximum length of mesoventrite.
6. Maximum length of metaventrite.
7. Relative area of lighter part of metafemur: measured flat in ventral view as the area of the lighter part of metafemur divided by the total area of the metafemur.
8. Maximum height of elytrum: measured in lateral view while barely seeing the opposite elytrum.
9. Pronotal Index (PI): measured as the length of the pronotum divided by the maximum width of the pronotum.
10. Pronotal-Elytral Index (PEI): measured as the length of the pronotum divided by the length of the elytra.
11. Ratio between length of elytra and width of pronotum: measured as the length of the elytra divided by the maximum width of the pronotum.

**Male genital characters**: all measurements in dorsal view. The mean of the left and right paramere was used as one character.

1. Area from tip of paramere to narrowest part of paramere.
2. Area of the most sclerotized darker part of penis.
3. Ratio between length and width of the darker part of sclerotized penis.
4. Ratio between upper and lower paramere lengths from the narrowest part of paramere: measured as length from bottom to narrowest part of paramere divided by length from tip to narrowest part of paramere.
5. Width of paramere at level where sclerotized part of penis has maximum width.
6. Ratio between upper and lower paramere lengths from character 5: measured as length from bottom of paramere to level at maximum width of sclerotized penis / length of paramere from maximum width of sclerotized penis to tip of paramere.
7. Length from tip of paramere to tip of penis: Measured as the length from the tip of the paramere to the tip of the sclerotized part of the penis.
8. Ratio RPP: Measured as the length of the paramere divided by the length from the tip of paramere to the tip of penis (character 7).
