# Supplementary material for: Species delimitation in northern European water scavenger beetles of the genus Hydrobius (Coleoptera, Hydrophilidae)
Source: Zookeys. 2016 Feb 16;(564):71–120. doi: 10.3897/zookeys.564.6558 (PMC4820092; doi:10.3897/zookeys.564.6558)

**Supplementary material 3: additional tables and figures**

**Table S1**. Borrowed specimens. An approximate number of examined specimens of the different morphotypes are given together with country.

| **Institution** | **Location (City, Country)** | ***H. f.* *fuscipes*** | ***H. f.* *rottenbergii*** | ***H. f.* *subrotundus*** | ***H. arcticus*** |
| --- | --- | --- | --- | --- | --- |
| Bavarian State Collection of Zoology (ZSM) | Munich, Germany | 3 Germany | 5 Germany | 1 Germany |  |
| Finnish Museum of Natural History (HELS) | Helsinki, Finland | 5 Finland | 9 Finland | 6 Finland | 7 Finland |
| Natural History Museum (UiO) | Oslo, Norway | 3 Norway | 7 Norway |  | 5 Norway |
| Natural History Museum (NHM) | London, UK | 1 China , 1 Israel, 1 Portugal, 1 Spain, 1 Turkey | 1 China, 1 Egypt, 6 France, 2 Hungary, 1 Spain, 1 Turkey, 4 UK | 6 UK |  |
| Natural History Museum of Denmark (COPEN) | Copenhagen, Denmark | 2 Denmark, 2 Greece, 1 Poland, 2 USA |  | 2 Denmark |  |
| Norwegian Institute for Nature Research (NINA) | Trondheim, Norway | 3 Norway |  | 3 Norway | 3 Norway |
| Senckenberg German Entomological Institute (SDEI) | Müncheberg, Germany |  | 2 Poland, 2 Germany | 2 Bulgaria |  |
| Tromsø University Museum (UiT) | Tromsø, Norway |  |  |  | 1 Norway |
| University Museum of Bergen (UiB) | Bergen, Norway |  | 2 Norway | 2 Norway | 6 Norway |

**Table S2**. Partition schemes and substitution models with the lowest Bayesian Information Criterion in PartitionFinder. Used in MrBayes and BEAST analyses. Abbreviations: G=gamma distribution, I = proportion of invariant sites, JC = Jukes Cantor ([Jukes and Cantor 1969](#_ENREF_3)), SYM = Symmetrical model ([Zharkikh 1994](#_ENREF_6)), F81 = Felsenstein 1981 ([Felsenstein 1981](#_ENREF_1)), HKY = Hasegawa-Kishino-Yano ([Hasegawa et al. 1985](#_ENREF_2)), K80 = Kimura 2-parameter ([Kimura 1980](#_ENREF_4)), TrN = Tamura Nei ([Tamura and Nei 1993](#_ENREF_5)), TrNef = TrN with equal base frequencies. Pos. = codon position.

| **Analyses used (software)** | **Gene segment** | **Subset Partitions** | **Best substitution model** |
| --- | --- | --- | --- |
| MrBayes with outgroup | COI | Pos. 1 | SYM+G |
|  |  | Pos. 2 | F81 |
|  |  | Pos. 3 | HKY+G |
|  | H3 | Pos. 1 and pos. 2 | JC+I |
|  |  | Pos. 3 | K80+I |
|  | ITS2 | - | HKY+G |
|  | Concatenated | COI pos. 1 and H3 pos. 1 | SYM+G |
|  |  | COI pos. 2 | F81 |
|  |  | COI pos. 3 | HKY+G |
|  |  | H3 pos. 2 | JC |
|  |  | H3 pos. 3 | K80 |
|  |  | ITS2 | HKY+I |
| BEAST without outgroup (strict clock) | COI | Pos. 1 | TrNef+I |
|  |  | Pos. 2 | HKY |
|  |  | Pos. 3 | TrN+G |
|  | H3 | Pos. 1 and pos. 2 | JC+I |
|  |  | Pos. 3 | K80 |
|  | ITS2 | - | HKY |

**Table S3.** Posterior probabilities of Clade I and Clade II as separate species under different prior combinations. Analyses conducted in BPP v3.0. Posterior probability values are the mean of multiple runs with different starting trees and algorithms (0 vs 1). Theta (*Θ*) = ancestral population size, tau (*τ*_0_) = age of root.

| ***A priori* assignment of Clade VII specimens** | **Parameter priors** (*Θ* , *τ*_0_) | | | |
| --- | --- | --- | --- | --- |
|  | G(1, 50), G(1, 20) | G(1, 50), G(1, 200) | G(1, 500), G(1, 20) | G(1, 500), G(1, 200) |
| As *H. arcticus* | 0.566 | 0.622 | 0.544 | 0.597 |
| As *H. f. rottenbergii* | 0.597 | 0.623 | 0.541 | 0.610 |
| As separate species | 0.590 | 0.616 | 0.545 | 0.602 |

**Table S4.** Posterior probabilities (PP) of species models from BPP v2.2. Based on multi-locus data (COI, H3 and ITS2) from 111 *Hydrobius* specimens. PP range from four prior-combinations. Models with PP < 0.05 are not reported. Species scenarios from Fig. S6.

| ***A priori* assignment of Clade VII specimens** | **Species model** | **Posterior probability** | | | | |
| --- | --- | --- | --- | --- | --- | --- |
|  |  | Scenario 1 | Scenario 2 | Scenario 3 | Scenario 4 | Scenario 5 |
| As *H. arcticus*/*H. f. rottenbergii* | 10 species, split between Clade I and Clade II | 0.40–0.45 | 0.39–0.45 | - | - | - |
|  | 11 species | 0.55–0.60 | 0.55–0.61 | - | - | - |
| As separate species | 11 species, split between Clade I and Clade II | - | - | 0.40–0.46 | 0.39–0.44 | 0.39–0.46 |
|  | 12 species | - | - | 0.54–0.60 | 0.55–0.60 | 0.54–0.61 |

**Table S5.** Effect of priors on split probabilities of (Clade I, Clade II) nodes in BPP v2.2. Theta (*Θ*) = ancestral population size, tau (*τ*_0_) = age of root. Species scenarios from Fig. S6.

| **Species scenario** | **Parameter priors** (*Θ* , *τ*_0_) | | | |
| --- | --- | --- | --- | --- |
|  | G(1, 50),  G(1, 20) | G(1, 50),  G(1, 200) | G(1, 500),  G(1, 20) | G(1, 500),  G(1, 200) |
| 1 | 0.57 | 0.60 | 0.55 | 0.59 |
| 2 | 0.55 | 0.61 | 0.55 | 0.60 |
| 3 | 0.56 | 0.60 | 0.54 | 0.60 |
| 4 | 0.56 | 0.60 | 0.56 | 0.59 |
| 5 | 0.57 | 0.59 | 0.54 | 0.61 |

**Table S6.** Mean ± SE and number of observations (n) of male genitalia characters of *Hydrobius* morphotypes. See material and methods for details on character measurements.

| **Character (unit)** | **Mean ± SE (n)** | | | |
| --- | --- | --- | --- | --- |
|  | *H. arcticus* | *H. f. rottenbergii* | *H. f. fuscipes* | *H. f. subrotundus* |
| Width of parameres, dorsal view (ln(µm)) | 3.98 ± 0.0157 (15) | 3.90 ± 0.0153 (15) | 3.74 ± 0.00754 (15) | 3.81 ± 0.00767 (16) |
| Robustness of parameres | 9.43 ± 0.154 (15) | 9.90 ± 0.242 (15) | 12.8 ± 0.180 (15) | 12.2 ± 0.158 (15) |
| Ratio paramere length / penis length | 1.47 ± 0.0180 (15) | 1.45 ± 0.0218 (15) | 1.58 ± 0.0139 (15) | 1.62 ± 0.0166 (15) |
| Width of parameres, lateral view (ln(µm)) | 3.79 ± 0.0343 (14) | 3.64 ± 0.0228 (15) | 3.71 ± 0.0166 (14) | 3.84 ± 0.0207 (16) |
| Curvature of paramere tip (µm) | 58.8 ± 2.13 (15) | 54.4 ± 1.33 (15) | 59.7 ± 1.83 (14) | 73.0 ± 1.61 (16) |

**Table S7.** Post hoc comparison of means of male genital characters in morphotypes of *Hydrobius*. Number of observations is approximately 15 of each morphotype (Table S6). t-value and corresponding adjusted p-value are used to see if differences between morphotypes are significantly different from 0. Adjusted p-values were calculated with Tukey’s HSD test. p-values lower than 0.05 in bold. See material and methods for details on character measurements.

| **Character (unit, degrees of freedom)** | **Morphotype** | **Morphotype** | **Difference between morphotypes ± SE** | **t-value** | **Adjusted p-value** |
| --- | --- | --- | --- | --- | --- |
| Width of parameres, dorsal view (ln(µm), df = 58) | *H. arcticus* | *H. f.* *rottenbergii* | 0.0889 ± 0.0172 | 5.17 | **< 0.001** |
|  | *H. arcticus* | *H. f.* *fuscipes* | 0.248 ± 0.0169 | 14.6 | **< 0.001** |
|  | *H. arcticus* | *H. f.* *subrotundus* | 0.172 ± 0.0169 | 10.2 | **< 0.001** |
|  | *H. f.* *fuscipes* | *H. f.* *rottenbergii* | 0.159 ± 0.0169 | 9.39 | **< 0.001** |
|  | *H. f.* *subrotundus* | *H. f.* *rottenbergii* | 0.0830 ± 0.0169 | 4.90 | **< 0.001** |
|  | *H. f.* *fuscipes* | *H. f.* *subrotundus* | 0.0760 ± 0.0167 | 4.56 | **< 0.001** |
| Robustness of parameres (df = 56) | *H. arcticus* | *H. f.* *rottenbergii* | 0.470 ± 0.265 | 1.78 | 0.294 |
|  | *H. arcticus* | *H. f.* *fuscipes* | 3.36 ± 0.265 | 12.7 | **< 0.001** |
|  | *H. arcticus* | *H. f.* *subrotundus* | 2.81 ± 0.265 | 10.6 | **< 0.001** |
|  | *H. f.* *fuscipes* | *H. f.* *rottenbergii* | 2.89 ± 0.265 | 10.9 | **< 0.001** |
|  | *H. f.* *subrotundus* | *H. f.* *rottenbergii* | 2.34 ± 0.265 | 8.86 | **< 0.001** |
|  | *H. f.* *fuscipes* | *H. f.* *subrotundus* | 0.542 ± 0.265 | 2.05 | 0.183 |
| Ratio paramere length / penis length (df = 56) | *H. arcticus* | *H. f.* *rottenbergii* | 0.0117 ± 0.0251 | 0.466 | 0.966 |
|  | *H. arcticus* | *H. f.* *fuscipes* | 0.113 ± 0.0251 | 4.48 | **< 0.001** |
|  | *H. arcticus* | *H. f.* *subrotundus* | 0.151 ± 0.0251 | 6.02 | **< 0.001** |
|  | *H. f.* *fuscipes* | *H. f.* *rottenbergii* | 0.124 ± 0.0251 | 4.95 | **< 0.001** |
|  | *H. f.* *subrotundus* | *H. f.* *rottenbergii* | 0.163 ± 0.0251 | 6.49 | **< 0.001** |
|  | *H. f.* *fuscipes* | *H. f.* *subrotundus* | 0.0387 ± 0.0251 | 1.54 | 0.420 |
| Width of parameres, lateral view (ln(µm), df = 55) | *H. arcticus* | *H. f.* *rottenbergii* | 0.160 ± 0.0365 | 4.38 | **< 0.001** |
|  | *H. arcticus* | *H. f.* *fuscipes* | 0.0843 ± 0.0371 | 2.27 | 0.118 |
|  | *H. arcticus* | *H. f.* *subrotundus* | 0.0414 ± 0.0360 | 1.15 | 0.660 |
|  | *H. f.* *fuscipes* | *H. f.* *rottenbergii* | 0.0755 ± 0.0365 | 2.07 | 0.177 |
|  | *H. f.* *subrotundus* | *H. f.* *rottenbergii* | 0.201 ± 0.0353 | 5.70 | **< 0.001** |
|  | *H. f.* *fuscipes* | *H. f.* *subrotundus* | 0.126 ± 0.0360 | 3.49 | **0.00516** |
| Curvature of paramere tip (µm, df = 56) | *H. arcticus* | *H. f.* *rottenbergii* | 4.47 ± 2.47 | 1.81 | 0.278 |
|  | *H. arcticus* | *H. f.* *fuscipes* | 0.854 ± 2.51 | 0.340 | 0.986 |
|  | *H. arcticus* | *H. f.* *subrotundus* | 14.2 ± 2.43 | 5.83 | **< 0.001** |
|  | *H. f.* *fuscipes* | *H. f.* *rottenbergii* | 5.32 ± 2.51 | 2.12 | 0.159 |
|  | *H. f.* *subrotundus* | *H. f.* *rottenbergii* | 18.6 ± 2.43 | 7.67 | **< 0.001** |
|  | *H. f.* *fuscipes* | *H. f.* *subrotundus* | 13.3 ± 2.47 | 5.38 | **< 0.001** |

**Table S8.** Intercept ± SE and number of observations (n) of morphological characters of *Hydrobius* morphotypes. Intercepts from linear regression models where the character was plotted against body size. See material and methods for details on character measurements.

| **Character (unit)** | **Intercept ± SE (n)** | | | |
| --- | --- | --- | --- | --- |
|  | *H. arcticus* | *H. f. rottenbergii* | *H. f. fuscipes* | *H. f. subrotundus* |
| Length of parameres (ln(µm)) | 5.71 ± 0.229 (15) | 5.65 ± 0.238 (15) | 5.73 ± 0.248 (15) | 5.77 ± 0.244 (15) |
| Elytral Index | 1.005 ± 0.0606 (21) | 1.076 ± 0.0649 (26) | 1.060 ± 0.0709 (33) | 1.003 ± 0.0678 (33) |

**Table S9.** Post hoc comparison of intercepts for length of parameres affected by body size. Length of parameres was plotted against body size in natural log-log scale for different morphotypes of *Hydrobius*. Number of observations: 15 of each morphotype. T-value and corresponding adjusted p-value are used to see if differences between morphotypes are significantly different from 0. Degrees of freedom = 55. Adjusted p-values were calculated with Tukey’s HSD test. p-values < 0.05 in bold.

| **Morphotype** | **Morphotype** | **Difference between morphotypes ± SE** | **t-value** | **Adjusted p-value** |
| --- | --- | --- | --- | --- |
| *H. arcticus* | *H. f.* *rottenbergii* | 0.061 ± 0.020 | 3.08 | **0.0158** |
| *H. arcticus* | *H. f.* *fuscipes* | 0.017 ± 0.026 | 0.635 | 0.918 |
| *H. arcticus* | *H. f.* *subrotundus* | 0.058 ± 0.023 | 2.49 | 0.0710 |
| *H. f.* *fuscipes* | *H. f.* *rottenbergii* | 0.078 ± 0.021 | 3.72 | **0.00250** |
| *H. f.* *subrotundus* | *H. f.* *rottenbergii* | 0.12 ± 0.019 | 6.23 | **< 0.001** |
| *H. f.* *fuscipes* | *H. f.* *subrotundus* | 0.041 ± 0.019 | 2.23 | 0.125 |

**Table S10.** Post hoc comparison of intercepts from a regression with Elytral Index affected by body size. Elytral Index was plotted against body size for different morphotypes of *Hydrobius*. Number of observations: 21 *H. arcticus,* 26 *H. f.* *rottenbergii,* 33 *H. f.* *fuscipes* and 33 *H. f.* *subrotundus*. T-value and corresponding adjusted p-value are used to see if differences between morphotypes are significantly different from 0. Degrees of freedom = 108. The adjusted p-values were calculated with Tukey’s HSD test. p-values < 0.05 in bold. See material and methods for details on character measurements.

| **Morphotype** | **Morphotype** | **Difference between morphotypes ± SE** | **t-value** | **Adjusted p-value** |
| --- | --- | --- | --- | --- |
| *H. arcticus* | *H. f.* *rottenbergii* | 0.070 ± 0.013 | 5.519 | **< 0.001** |
| *H. arcticus* | *H. f.* *fuscipes* | 0.055 ± 0.016 | 3.544 | **0.00314** |
| *H. arcticus* | *H. f.* *subrotundus* | 0.0017 ± 0.014 | 0.124 | 0.999 |
| *H. f.* *fuscipes* | *H. f.* *rottenbergii* | 0.015 ± 0.012 | 1.236 | 0.598 |
| *H. f.* *subrotundus* | *H. f.* *rottenbergii* | 0.072 ± 0.011 | 6.485 | **< 0.001** |
| *H. f.* *fuscipes* | *H. f.* *subrotundus* | 0.057 ± 0.011 | 5.387 | **< 0.001** |

**References**

Felsenstein J (1981) Evolutionary trees from DNA sequences: a maximum likelihood approach. Journal of Molecular Evolution 17: 368-376. doi:10.1007/bf01734359

Hasegawa M, Kishino H, Yano T-a (1985) Dating of the human-ape splitting by a molecular clock of mitochondrial DNA. Journal of Molecular Evolution 22: 160-174. doi:10.1007/bf02101694

Jukes TH, Cantor CR (1969) Evolution of Protein Molecules. Mammalian Protein Metabolism: 21-132. doi:10.1016/b978-1-4832-3211-9.50009-7

Kimura M (1980) A simple method for estimating evolutionary rates of base substitutions through comparative studies of nucleotide sequences. Journal of Molecular Evolution 16: 111-120. doi:10.1007/bf01731581

Tamura K, Nei M (1993) Estimation of the number of nucleotide substitutions in the control region of mitochondrial DNA in humans and chimpanzees. Molecular Biology and Evolution 10: 512-526. DOI:

Zharkikh A (1994) Estimation of evolutionary distances between nucleotide sequences. Journal of Molecular Evolution 39: 315-329. doi:10.1007/bf00160155

Figure legends

**Figure S1.** Majority-rule consensus tree from Bayesian analysis of COI sequences. Branch support values are posterior probabilities. Samples are labeled with ID-numbers, identified morphotypes and country of origin. Specimens collected in sympatry are also labeled with locality name (Rinnleiret, Motzen or Oland). Samples from BOLD labeled with Sequence ID from BOLD. Scale bar indicates expected number of nucleotide substitutions per site. Abbreviations of morphotypes: arc=*arcticus*, fus=*fuscipes*, rot=*rottenbergii*, sub=*subrotundus*.


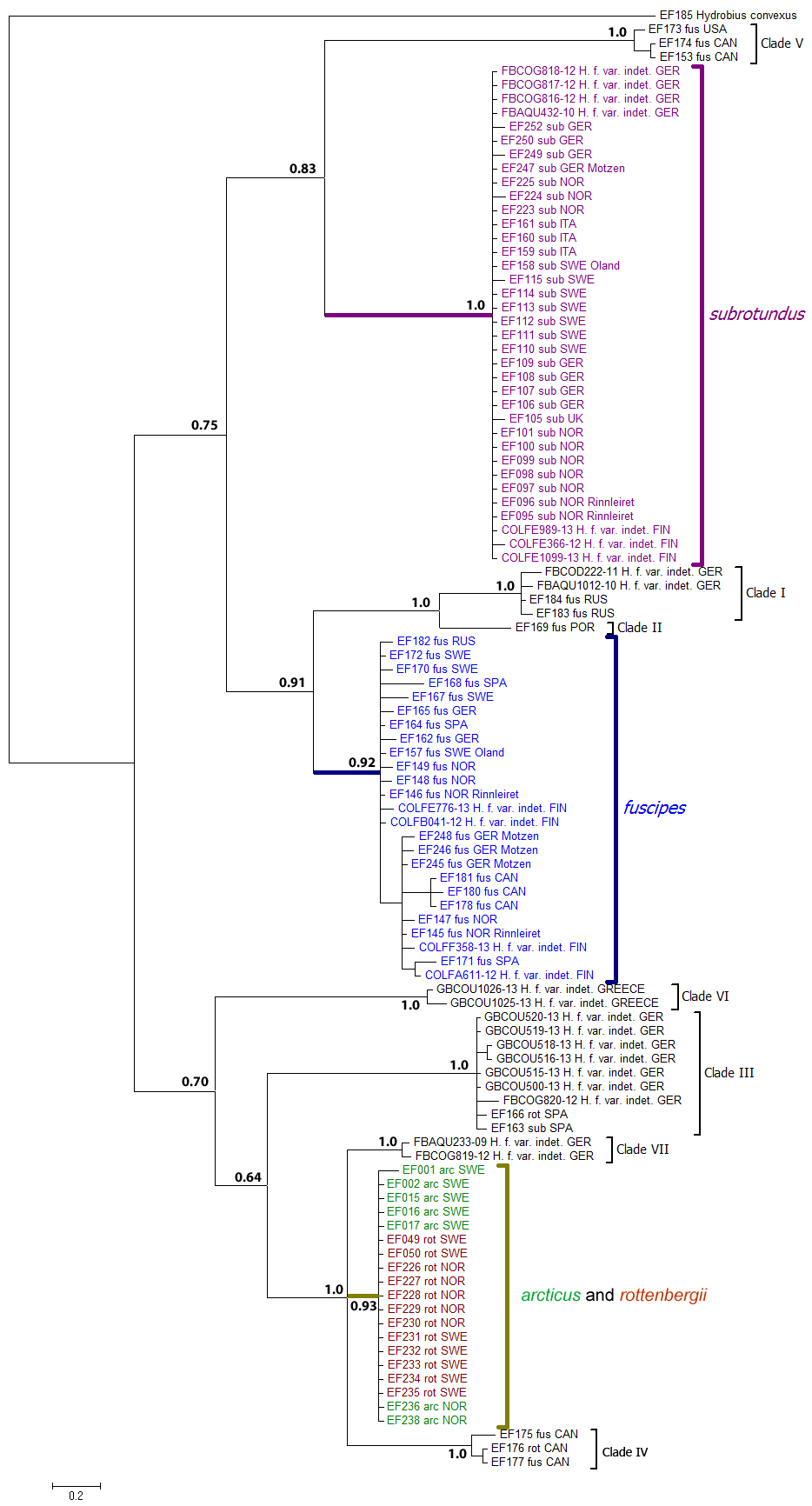


**Figure S2.** Majority-rule consensus tree from Bayesian analysis of H3 sequences. Branch support values are posterior probabilities. Terminal names and abbreviations as in Fig. S1. Scale bar indicates expected number of nucleotide substitutions per site. Branches with “//” have been manually cut.

**
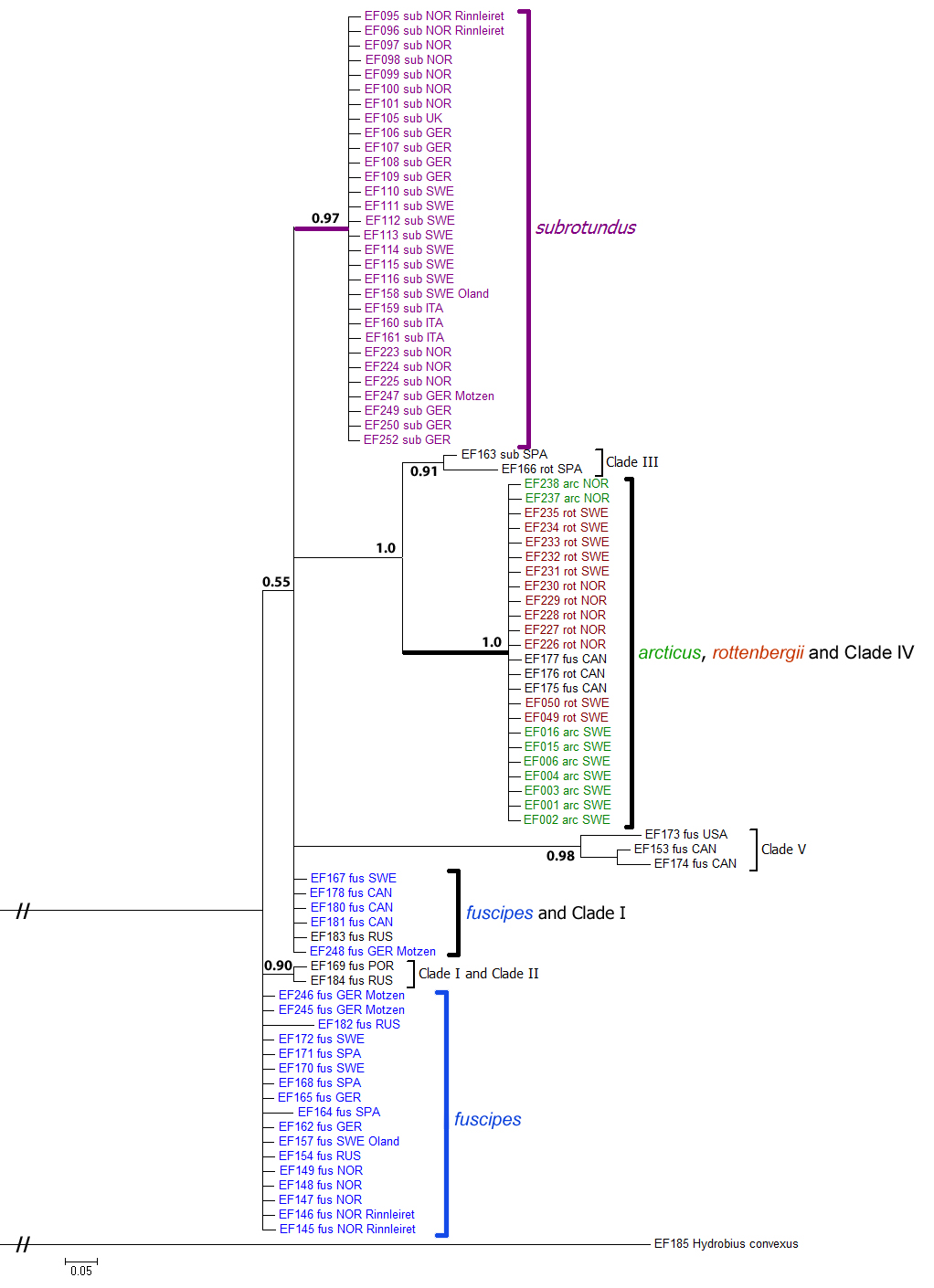
**

**Figure S3.** Majority-rule consensus tree from Bayesian analysis of ITS2 sequences. Branch support values are posterior probabilities. Terminal names and abbreviations as in Fig. S1. Scale bar indicates expected number of nucleotide substitutions per site.


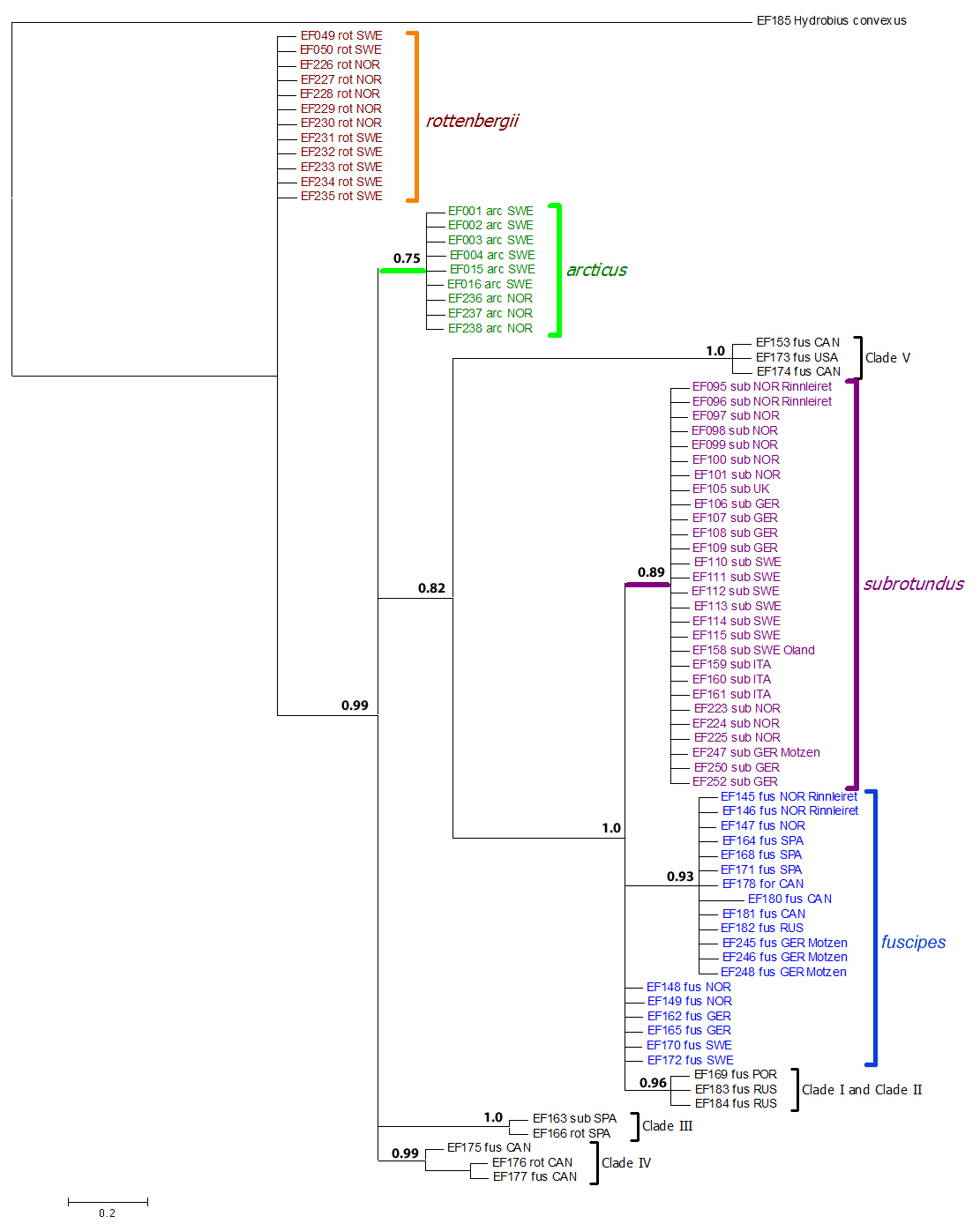


**Figure S4.** Ultrametric (strict clock) maximum clade credibility (MCC) tree used in GMYC analysis of H3. Terminal names and abbreviations as in Fig. S1. Values next to branches show Bayesian posterior probability support (node values with PP < 0.5 not shown). Scale bar represents an artificial time scale with the root at time 1.


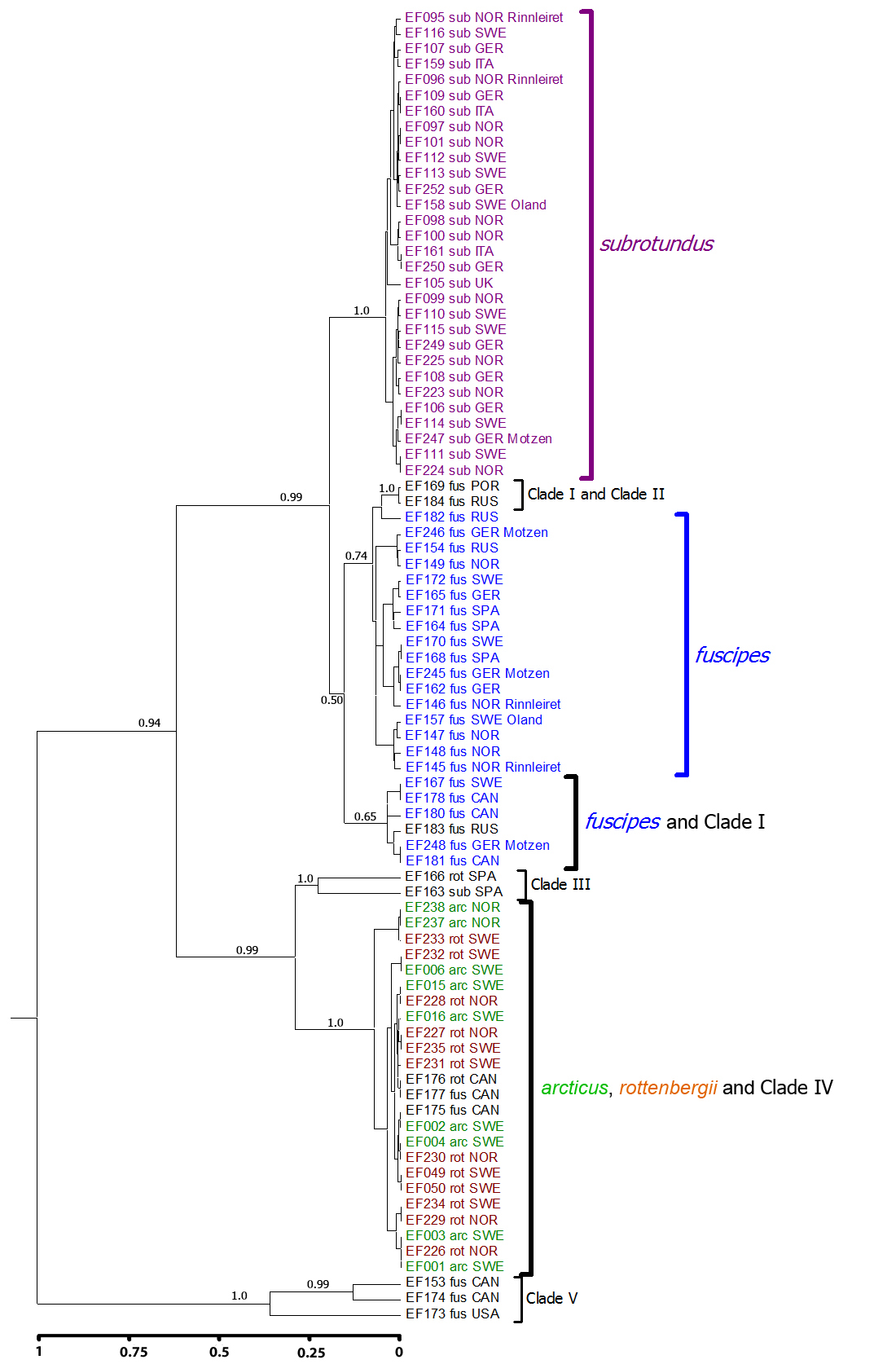


**Figure S5.** Species trees with highest posterior probability from BPP v3.0 analyses conducted on 111 *Hydrobius* specimens. Multi-locus data (COI, H3 and ITS2) used with *H. convexus* included as outgroup. **a)** Species tree with largest posterior probability (PP). **b)** Species tree with second largest PP. **c-d)** Subtrees from node marked “X” in **a** and **b**. **c)** Tree with 12 species delimited (including outgroup). **d)** Tree with 11 species delimited, Clade I and Clade II considered the same species. * Clade VII only delimited when specimens from Clade VII were *a priori* assigned as a potential species separate from *H. arcticus* and *H. f. rottenbergii*.

**
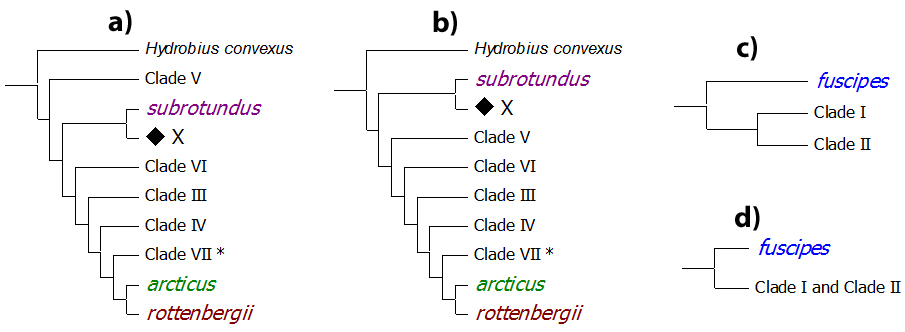
**

**Figure S6.** Results of BPP v2.2 analyses conducted on 111 *Hydrobius* specimens. Multi-locus data (COI, H3 and ITS2) used with *H. convexus* as outgroup. A) Guide tree. B-E) subtrees from node marked “X” in A, used in 5 different species scenarios. B) Topology of species scenario 1 and 2. Specimens of Clade VII *a priori* assigned as *H. arcticus* in “species scenario 1” and as *H. f. rottenbergii* in “species scenario 2”. C) Topology of species scenario 3. D) Topology of species scenario 4. E) Topology of species scenario 5. Values next to branches indicate range of split posterior probabilities, i.e. the probability for the node representing a speciation event, from four different prior-combinations that were used in each of the species scenarios. Values in red have split probabilities < 0.9. Samples were assigned to potential species groups based on genetically divergent clades in Table 5, taxonomic knowledge and GMYC-delimited species.


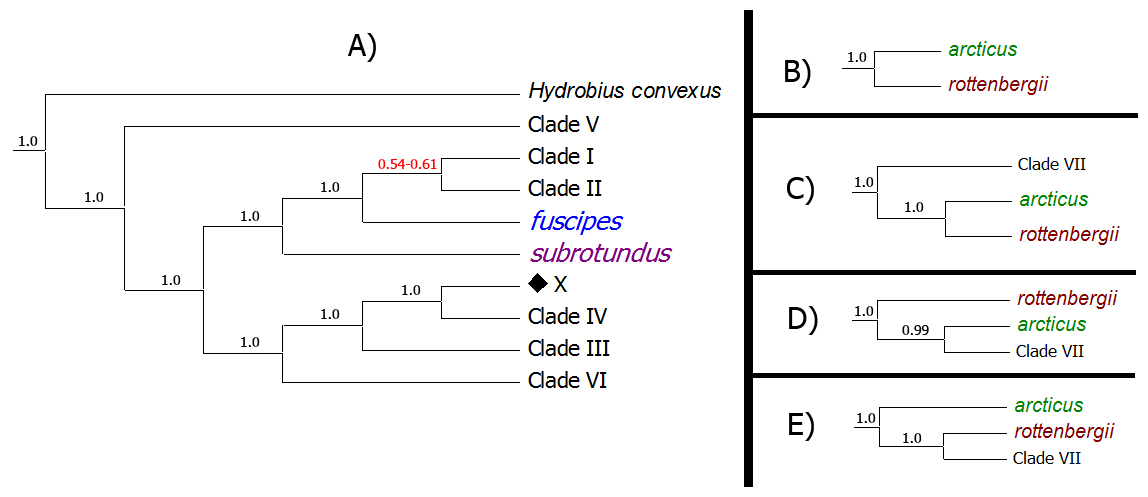

Supplement: Supplementary material 3 — Supplementary tables and figures [file zookeys-564-071-s003.docx]
